# Recent Advances in the Field of Amino Acid-Conjugated Aminoferrocenes—A Personal Perspective

**DOI:** 10.3390/ijms25094810

**Published:** 2024-04-28

**Authors:** Mojca Čakić Semenčić, Monika Kovačević, Lidija Barišić

**Affiliations:** Department of Chemistry and Biochemistry, Faculty of Food Technology and Biotechnology, University of Zagreb, 10000 Zagreb, Croatia; mojca.cakic@pbf.unizg.hr (M.Č.S.); monika.kovacevic@pbf.unizg.hr (M.K.)

**Keywords:** biological activity, chirality, chiroptical probe, conformational analysis, density functional theory (DFT), ferrocene, helicity, hydrogen bonds, mimetic, turn structure

## Abstract

The development of turn-based inhibitors of protein–protein interactions has attracted considerable attention in medicinal chemistry. Our group has synthesized a series of peptides derived from an amino-functionalized ferrocene to investigate their potential to mimic protein turn structures. Detailed DFT and spectroscopic studies (IR, NMR, CD) have shown that, for peptides, the backbone chirality and bulkiness of the amino acid side chains determine the hydrogen-bond pattern, allowing tuning of the size of the preferred hydrogen-bonded ring in turn-folded structures. However, their biological potential is more dependent on their lipophilicity. In addition, our pioneering work on the chiroptical properties of aminoferrocene-containing peptides enables the correlation of their geometry with the sign of the CD signal in the absorption region of the ferrocene chromophore. These studies have opened up the possibility of using aminoferrocene and its derivatives as chirooptical probes for the determination of various chirality elements, such as the central chirality of amino acids and the helicity of peptide sequences.

## 1. Introduction

**Figure 1:**
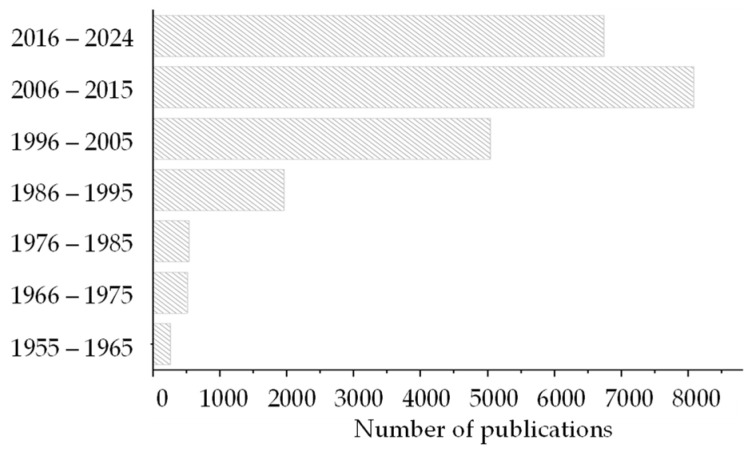
Number of publications per decade from a Web of Science search for articles with topics relating to ferrocene, taken 24 January 2024.

It appears that ferrocene was unexpectedly produced during the conversion of dicyclopentadiene to cyclopentadiene in the late 1940s. However, the first reports [1,2], by two different groups, caused a great deal of controversy, because until then no stable molecule consisting of only a hydrocarbon and a transition metal was known. The unusual stability of ferrocene results from the presence of two aromatic cyclopentadienyl ions, C5H5^−^, with ten electrons from five carbon–carbon single bonds and a cloud of six additional electrons located above and below the plane of the aromatic rings. This similarity to benzene prompted chemists to call it ferrocene [3]. Due to its exceptional properties (stability, electrophilic and redox reactivity, good solubility in organic solvents, and stability in air), ferrocene has established itself as an icon of organometallic chemistry, and its derivatives have been reported in more than 23,000 publications (Web of Science, Figure 1).

A considerable number of compounds derived from ferrocenes functionalized with reactive groups tethered to one or both cyclopentadienyl rings have been synthesized to date, and a comprehensive overview of their application in a variety of fields, including catalysis, materials science and sensing, and medicine, has recently been given [4,5,6,7,8]. In addition, our work on the conformational and biological evaluation of ferrocene conjugates with mannose, resveratrol, and natural amino acids was systematically reviewed [9].

It is known that ferrocenes play an important role in medicinal chemistry: (i) as a bulky isostere that can fit tightly into a hydrophobic pocket of a target, enhancing its binding affinity [10], (ii) as a redox mediator that can promote the conversion of an inactive prodrug into an active drug, as found for the ferrocene analogue of tamoxifen [11], and (iii) as a ROS generator in biological media, as demonstrated for the ferrocene analogue of chloroquine [12].

Amino-functionalized ferrocenes have attracted much attention due to their application in the areas of electroactive indicators, biosensors and biofuel cells, the bioisosteric modification of antiparasitic and anticancer drugs, peptide bioconjugates and prodrugs, etc. A detailed review on the synthesis, reactivity, and application of aminoferrocene and its derivatives in areas of redox-active dendrimers, prodrugs, metalloligands, and the sensing of biomolecules and anions was published in 2016 [13]. The more recent contributions regarding the electroactive aminoferrocene-containing compounds relate to their use as electroactive redox probes for the detection of: hypochlorous acid in living systems and food samples [14], alkaline phosphatase activity as a biomarker for the clinical diagnosis of bone and hepatobiliary diseases [15], *Salmonella* contamination in food and water [16], tumor marker alpha fetoprotein [17], etc. In addition, recent advances in ferrocene-based electrocatalytic systems in various electrochemical fields have been summarized [18]. Aminoferrocenes have been shown to transform to aminoferrocenium ions under oxidative conditions, after which they are degraded to free iron ions, an efficient catalyst for ROS production and trigger of oxidative stress and cell death [19]. Further progress in the development of anticancer aminoferrocene prodrugs was systematically reviewed in 2019 [20]. *N*-alkylaminoferrocene-based prodrugs, activated under cancer-specific conditions, i.e., a high ROS concentration, were able to reduce the mitochondrial membrane potential, but also showed low efficacy and high toxicity [21,22,23]. Structural optimization led to aminoferrocene derivatives that are able to generate mitochondrial ROSs only in cancer cells, and therefore have anticancer activity in vivo [24]. It has been shown that the self-immolative amphiphilic poly(ferrocenes) containing aminoferrocene side chains are triggered by acidic tumor conditions; the aminoferrocene moiety reacts to give Fe^2+^, which can catalyze the conversion of intracellular H_2_O_2_ to highly reactive ^˙^OH, leading to the oxidative stress of tumor cells [25]. In addition, an isosteric aminoferrocene derivative of regorafenib, an FDA-approved drug for the treatment of metastatic colorectal cancer, has been reported to have promising efficacy against cancer cell lines [26].

Our previous overview of homo- and hetero-chiral aminoferrocene conjugates (including Boc–l–Pro–l–Ala–**NH**–**Fn**, Boc–d–Pro–d–Ala–**NH**–**Fn**, Boc–l–Pro–d–Ala–**NH**–**Fn**, Boc–d–Pro–l–Ala–**NH**–**Fn** [27], Ac–Ala–**NH**–**Fn**–**NH**–Ala–Boc, Ac–Ala–**NH**–**Fn**–**NH**–Ala–Ac [28] and Ac–l–Ala–l–Pro–**NH**–**Fn**–**NH**–l–Pro–l–Ala–Boc, Ac–d–Ala–l–Pro–**NH**–**Fn**–**NH**–l–Pro–d–Ala–Boc, Ac–l–Ala–l–Pro–**NH**–**Fn**–**NH**–l–Pro–l–Ala–Ac, and Ac–d–Ala–l–Pro–**NH**–**Fn**–**NH**–l–Pro–d–Ala–Ac [29]) was given in 2017 [9]. These peptides were subjected to detailed conformational analysis in solution and in the solid state to determine the hydrogen bonding pattern within the same or between podand peptide strands, i.e., to determine the most commonly adopted secondary structural element. In addition, their antitumor activity was mainly evaluated as low or moderate.

Furthermore, we linked two amino-functionalized ferrocenes via an oxalamide bridge to form MeOOC–**Fn**–**NH**–CO–CO–**NH**–**Fn**–COOMe [30]. A conformational analysis of this dimer revealed a pattern based on NH_Fn_⋅⋅⋅OC_COOMe_ intramolecular hydrogen bonds (IHBs). Although most of the criteria for the formation of a good gelator (solubility, presence of hydrophilic (oxalamide) and hydrophobic (ferrocene) moieties, chirality [31]) were met, the gelation potential was rather poor.

**Figure 2:**
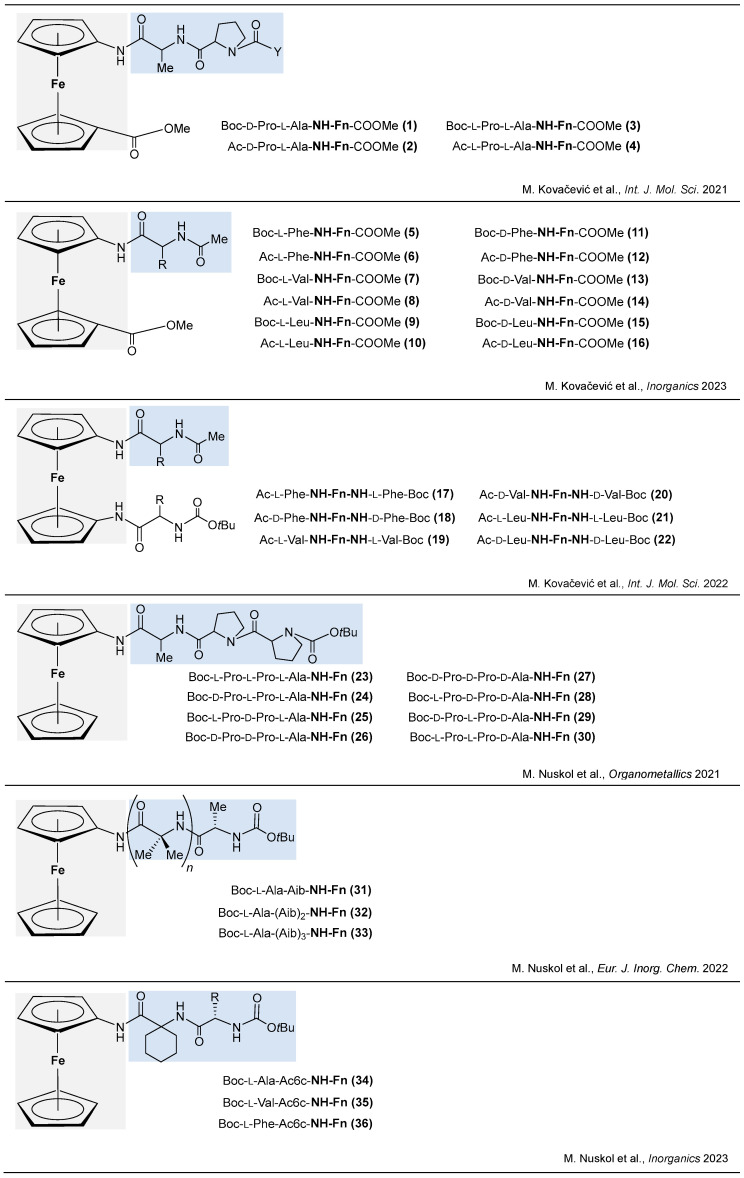

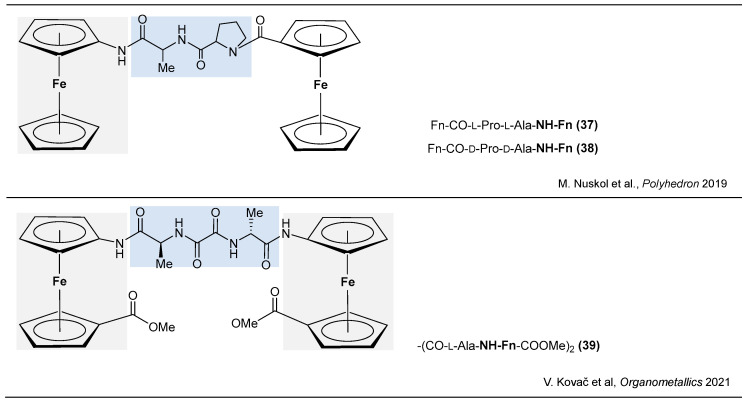
The conjugates of amino-functionalized ferrocene and amino acids **1**–**39** [32,33,34,35,36,37,38,39].

The aim of this review is to highlight our work over the last five years on the structure and activity of amino-functionalized ferrocene conjugates with amino acids and peptides (Figure 2). As far as we know, the conformational and biological properties of aminoferrocene-containing peptides have not yet been studied by other authors.

**Scheme 1:**
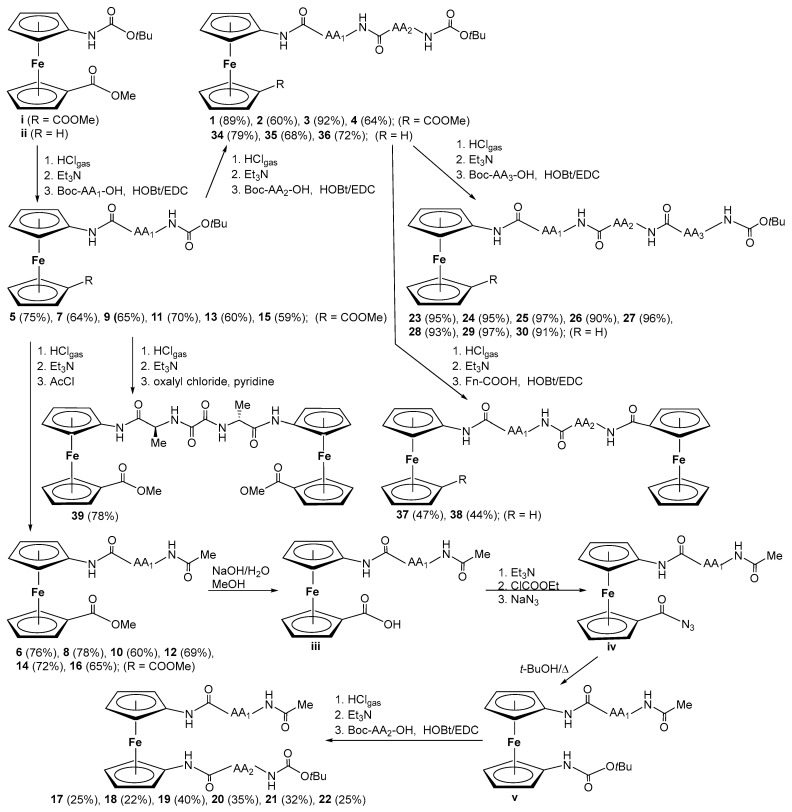
Synthesis of α-amino acid derivatives **1**–**30** and **34**–**39**.

The α-amino acid derivatives **1**–**30** and **34**–**39** were prepared from Boc–NH–Fn–R (**i**, R = COOMe [40]; **ii**, R = H [41]) according to the synthesis procedure established in our group (Scheme 1). In general, the elongation of the *N*-terminus of ferrocene precursors involves two steps: (i) Boc-deprotection in the presence of gaseous HCl to give hydrochloride salt, which is then treated with an excess of triethylamine (NEt_3_) to afford the free amine, and (ii) hydroxybenzotriazole (HOBt)/ethyl-(*N′*,*N′*-dimethylamino)propylcarbodiimide hydrochloride (EDC) coupling of the free amine with *C*-activated Boc−AA−OH or Fn–COOH, respectively, to obtain the unsymmetrically substituted peptides **1**–**4**, **5**, **7**, **9**, **11**, **13**, **15**, **23**–**30**, and **34**–**38**. In addition, oxalamide **39** was prepared by treating ferrocene amine with oxalyl chloride.

To obtain the symmetrically disubstituted and orthogonally protected conjugates **17**–**22**, the Boc-groups of their carbamate precursors were cleaved in acidic milieu and converted to acetamides **6**, **8**, **10**, **12**, **14**, and **16**. Then, the ester groups attached to the lower cyclopentadiene rings were hydrolyzed to give acids **iv**; an equimolar amount of NaOH was used to prevent the racemization, and saponification was carried out at 80 °C for 1 h. The obtained acids were converted to unstable azides **v**, followed by an in situ Curtius rearrangement to carbamates **vi**. To prevent the undesired conversion of the intermediate isocyanate group to *sym*-urea, the *t-*BuOH was freshly dried, and the reaction temperature was maintained at 65 °C. The conversion of azide (~2135 cm^−1^) to carbamate via isocyanate (~2270 cm^−1^) was completed when its IR absorption bands disappeared. The acidic Boc-deprotection and coupling with *C*-activated Boc−AA−OH gave the goal peptides **17**–**22**.

**Scheme 2:**
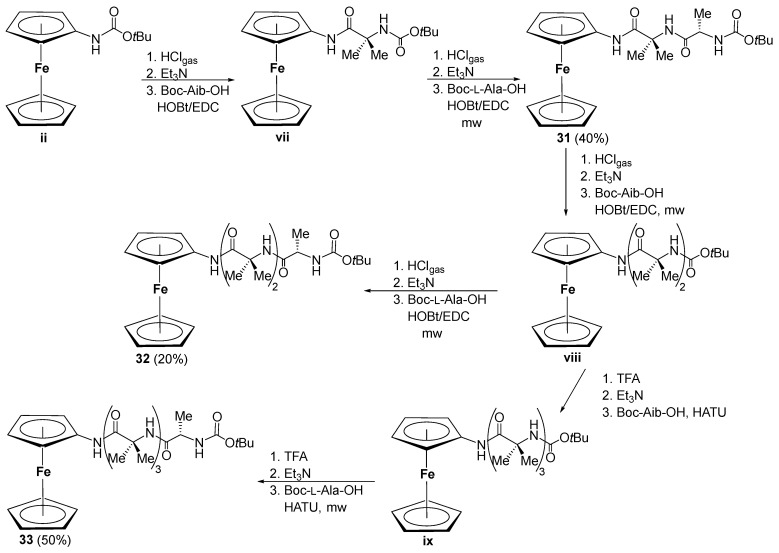
Synthesis of α,α-amino acid (Aib) derivatives **31**–**33**.

The synthesis of peptides **31**–**33** was microwave-assisted and is given in Scheme 2. Compared to the coupling of α-amino acids (Scheme 1), the synthesis involving α,α-amino acids such as 2-aminoisobutyric acid (Aib) proved to be much more challenging (Scheme 2) and required the use of different coupling methods. The standard HCl deprotection of Boc-aminoferrocene **ii** and EDC/HOBt-mediated copulation with free acid gave 85% of the lowest homolog of Aib peptide **vii**, but only 22% of **31** and 25% of **vii** were obtained under the same conditions. These yields were doubled to 40% and 54%, respectively, by exposing the reaction mixture to microwaves at *T* = 55 °C (*P* = 200 W) for 35 min. Under the same reaction conditions (HOBt/EDC, mw), only 20% of the target compound **32** was obtained, probably due to the prolonged deprotection of **iii** with HCl_gaseous_, which led to the decomposition of the reactant. To avoid the same problem in the synthesis of **viii**, the Boc functional group of **vii** was cleaved with trifluoroacetic acid (TFA) and the free amine was coupled with Boc–Aib–OH that was previously activated with 1-[bis(dimethylamino)methylene]-1*H*-1,2,3-triazolo [4,5-b]pyridinium 3-oxid hexafluorophosphate (HATU), which gave 49% of Aib-tripeptide **ix** after 20 h of stirring at room temperature. Conjugate **33** was prepared under the same reaction conditions (TFA cleavage, HATU activation) but with microwave exposure (2 h, *T* = 55 °C, *P* = 200 W), which gave a 50% yield.

## 2. The Turn-Inducing Capacity and Biological Activity of Conjugates 1–22

Protein–protein interactions (PPIs) are critical for most biological processes [42,43], and are undertaken by mechanisms involving small “hot spots”, hydrophobic structural regions containing from four to eight amino acids arranged as α-helices, β-sheets, or turns [44]. As disorders in PPIs can trigger diseases, the inhibitors capable of mimicing these recognition motifs at the PPI’s interface [45,46], including peptidomimetic-based inhibitors, have been investigated for their drug-like properties [47,48]. Peptidomimetics are compounds whose essential pharmacophoric units mimic the structural elements of a natural peptide or protein to retain their ability to recognize and bind to a biological target, thereby achieving the desired biological effect [49]. The transforming of the peptides to peptidomimetics provides low-side-effect compounds with improved proteolytic stability and absorption, and thus represents an efficient approach in medicinal chemistry [50]. The folding of peptidomimetics into predictable secondary structures also contributes to their drug-like properties [51,52,53]. Turns are usually located on the protein surface, where they are exposed to cell receptors and therefore serve as sites for molecular recognition [54,55]. They are formed when the polypeptide chain folds and reverses its direction by almost 180°, making the proteins compact and globular Taking into account their length and hydrogen bonding motif, turns are classified as α- (13-membered hydrogen-bonded (HB) ring), β- (10-membered HB ring), and γ-turns (7-membered HB ring). One of the design approaches in the field of turn mimetics is based on the turn-inducing element, which, in addition to inducing the turn as a secondary structural element, also restricts the conformational flexibility upon incorporation into the peptide chain. The role of ferrocene scaffolds as turn-inducing elements has been extensively studied in our group. We have shown that mono- and 1,1′-disubstituted ferrocene scaffolds equipped with the –CO group as a hydrogen bond acceptor and/or the –NH group as a hydrogen bond-donating site are able to induce the formation of turn- and β-sheet-like structures upon conjugation with amino acids [9,28,29,56,57]. A detailed conformational analysis combining theoretical (DFT) and experimental methods (IR, NMR, and CD spectroscopy) revealed that the conformational patterning of ferrocene peptides is regulated by the hydrogen bond-donating/-accepting properties of the ferrocene scaffolds, the steric hindrance derived from the amino acid side chains, and the bulkiness and basicity of the *N*-protecting groups, but is also strongly influenced by the chirality of the peptide backbone. Considering the number of ferrocene conjugates tested so far for the treatment of infectious diseases and the need to develop new bioactive ferrocene conjugates with drug-like properties, we tested the antitumor, antimicrobial, and antioxidant potentials of conjugates **1**–**22**.

### 2.1. Conjugates of Amino-Functionalized Ferrocene with Homo- and Heterochiral Pro–Ala Dipeptides ***1**–**4*** [32]

In general, the Pro-Xaa sequence is known as a β-turn-inducing motif [58,59,60]. Although we have expected that the bioconjugates **1**–**4** will adopt β-turn structures based on 10-membered HB rings, we wanted to test the influence of the chirality of Pro–Ala sequences on the stability of the conformations obtained. The conformational analysis involves a joint computational and experimental study.

#### 2.1.1. Computational Study

The theoretical conformational analysis of all derivatives described in this work comprises three steps. Using molecular mechanics (force field OPLS2005), a series of optimized low-level geometries were determined using the algorithms available in MacroModel [61,62]. The most stable geometries were then optimized at the B3LYP/LanL2DZ level of theory in Gaussian16 [63]. Finally, the most stable conformers were re-optimized at the B3LYP/6-311+G(d,p) level of theory (LanL2DZ basis set on Fe), where the solvent (chloroform) was modeled as a polarizable continuum (IEF-PCM) [64]. The relative energies reported in this work refer to the standard Gibbs free energies at 298 K. The quantum theory of atoms in molecules (QTAIM) was used to characterize the hydrogen bonds according to the Koch and Popelier criteria [65,66]. Theoretical CD spectra were calculated using time-dependent (TD)-DFT calculations.

**Figure 3:**
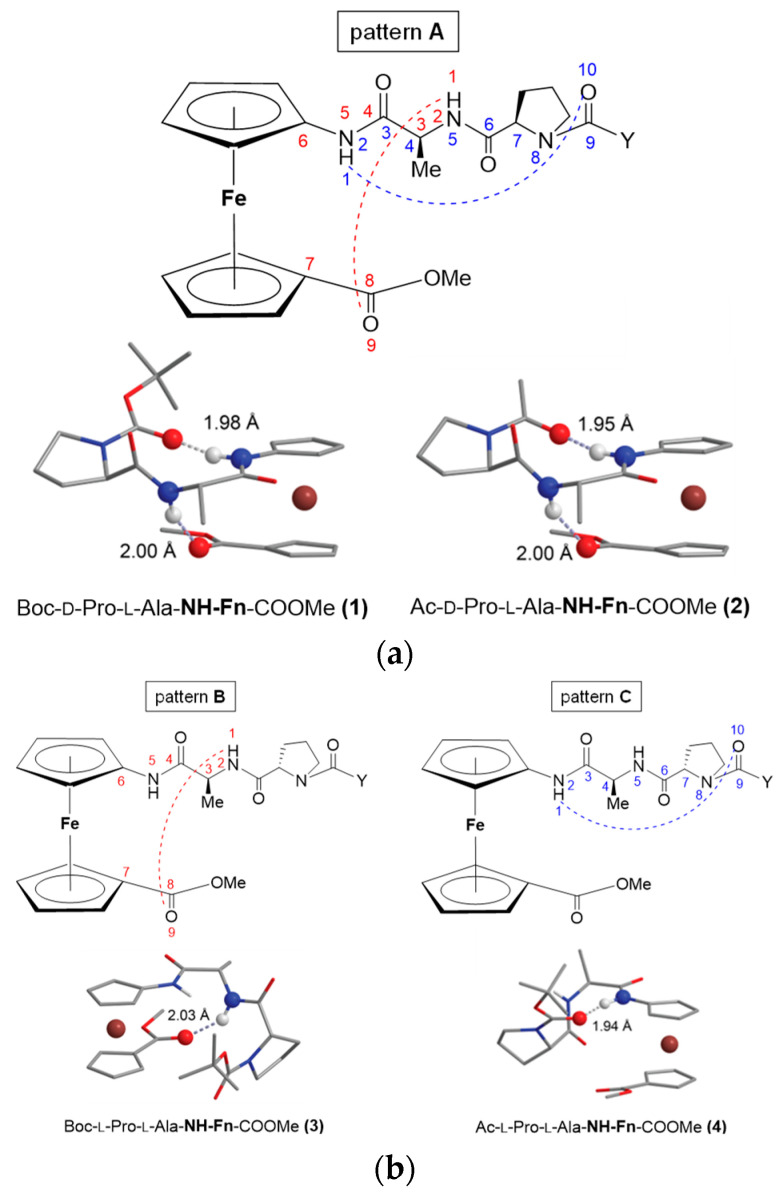
The DFT-predicted HB patterns in (**a**) hetero- (**1** and **2**) and (**b**) homo-chiral conjugates (**3** and **4**). The 3D conformations are adopted from reference [32].

The results of the computational study suggest that the heterochiral conjugates **1** and **2** adopt the rigid HB pattern **A** based on an intrastrand NH_Fn_···O=C_Boc/Ac_ HB corresponding to β-turn, and an additional interstrand NH_Ala_···O=C_COOMe_ HB forming a nine-membered ring (Figure 3a). However, the homochirality contributes to the conformational flexibility, as one of these HBs (pattern **B** or **C**) is mostly observed in conjugates **3** and **4** (Figure 3b).

It is expected that the computationally predicted conformations will be confirmed by experimental data.

#### 2.1.2. Spectroscopic Study

The experimental conformational analysis of peptidomimetics in solution is performed by combining the results of IR, NMR, and CD spectroscopy, which should show good agreement [67]. Once, when the most stable HB pattern was predicted by DFT, the NH region in the IR spectra was examined to determine the proportion of hydrogen-bonded states. The NMR spectra were then measured to specify which NH groups were involved in the HBs and how strong they were. When the hydrogen-bonded states prevailed and the HBs were found to be stronger, the Cotton effects in the CD spectra were more intense, indicating the presence of a highly ordered structure. To avoid the limitations and potential weaknesses of the individual spectroscopic methods, they were used in combination with the aim of clearly confirming the results of each individual method.

IR spectroscopy is a valuable tool for studying the secondary structure, conformational changes, structural dynamics, and stability of polypeptides and proteins. This method is convenient, non-destructive, requires a small sample, and is applicable under various conditions. Hydrogen bonding not only stabilizes the structure, but also lowers the frequency of the NH and CO absorption bands [68]. NMR spectroscopy is a powerful, non-invasive, and routine method of studying the structure and interactions of peptides and proteins and allows for the identification of bioactive conformations related to their drug-like properties. Recently, an overview of NMR methods for obtaining the 3D structure of small peptides was given [69]. Taking into account peptides’ conformational flexibility, the advantage of IR spectroscopy is reflected in its ability to recognize most of the states that interconvert on fast time scales. Therefore, the observed multiple distinct absorption bands belonging to the same vibration are attributed to the presence of multiple conformations. However, due to the slow NMR time scale and rapid interconversion of different conformations, the coalescence of their signals is observed [70,71].

The details of the spectroscopic techniques are not listed here so as not to overload the paper. They can be found in the extensive supplementary materials provided together with the cited articles [32,33,34,35,36,37,38,39].

1. IR Spectroscopy

The N–H···O=C HBs, predicted by DFT calculations, should cause a red shifting and increasing intensity in the N–H stretching vibrations [72,73]. As can be seen in Table 1, there are two distinct absorptions in the amide A region corresponding to free (~3420 cm^−1^) and associated (~3300–3325 cm^−1^) NH groups. Looking at the ratio of the intensities of the free and associated NH bands, it can be seen that the heterochiral conjugates **1** and **2** (*I*_NHfree_/*I*_NHassoc._~1/2.4) are more strongly involved in hydrogen bonding compared to their homochiral counterparts **3** and **4** (*I*_NHfree_/*I*_NHassoc._~1/1.5). The results obtained corroborate DFT-indicated involvement of the heterochiral peptides in more stable IHB-based conformations.

2. Concentration-dependent IR Spectroscopy

Since the β-turn structures are based on IHBs, we measured IR spectra at different dilutions (from 5 × 10^−2^ M to 1.25 × 10^−2^ M). Since the ratios of free and associated NH bands did not change, the involvement of the tested peptides in IHBs was strongly suggested. Otherwise, peptide aggregation would be reflected by a decrease in the intensity of the intermolecularly engaged NH bands upon dilution compared to the free NH bands.

3. NMR Spectroscopy

In protein chemistry, NMR spectroscopy is used to study the structures and conformations related to drug-like activity [69]. Therefore, the bioconjugates **1**–**4** were subjected to detailed 1D (^1^H, ^13^C) and 2D NMR studies (^1^H-^1^H COSY, ^1^H-^1^H NOESY, ^1^H-^13^C HMQC, and ^1^H-^13^C HMBC) to assign their structures and determine the individual hydrogen bonds and their strength as well as the hydrogen bonding patterns. Due to the deshielding effect of hydrogen bonding, the ^1^H chemical shifts of the involved amide protons move downfield (*δ* > 7 ppm), and the stronger hydrogen bonding leads to higher values of the chemical shifts [74]. Thus, the significantly downfield-shifted NH_Fn_ peaks (*δ* ~ 8–8.6 ppm) compared to NH_Ala_ (*δ* ~ 6.9–7.2 ppm) suggest their participation in stronger HBs (Table 2).

To further determine the conformational properties of the tested peptides, we measured the concentration, temperature, and solvent dependencies of the NMR chemical shifts.

4. Concentration-dependent NMR Spectroscopy

Since no significant upfield shifts of NH_Fn_ or NH_Ala_ were observed when measuring the NMR spectra during dilution from 50 mM to 1 mM, the IHB engagement was indicated, confirming the concentration-independent IR data.

5. Solvent-dependent NMR Spectroscopy

In addition to the hydrophobic effect, Coulomb interactions, and van der Waals interactions, IHBs also control protein folding [75]. When the IHBs are weak, the amide NH protons involved are exposed to a strong hydrogen-bond acceptor such as DMSO and their resonances are moved downfield. Otherwise, the amide NH protons engaged in a strong IHB are shielded from solvation and no significant change in their chemical shift (Δ*δ*) is observed during titration with dimethyl sulfoxide (DMSO). Therefore, the pronounced solvent dependence of the NH_Ala_ of the tested peptides (Δ*δ*~0.94–1.46 ppm) is attributed to their involvement in weak HBs. As for the solvent-sensitivity of NH_Fn_, increasing the DMSO content from 0% to 56% had a less pronounced effect on the NH_Fn_ of heterochiral peptides **1** and **2** (Δ*δ*~0.3–0.45 ppm) than on that of homochiral analogues **3** and **4** (Δ*δ*~1 ppm), providing further evidence that heterochirality contributes to participation in stronger IHBs and folding into more stable turn structures (Table 2).

6. Temperature-dependent NMR Spectroscopy

The temperature coefficients (Δ*δ*/Δ*T*) reflect the changes in the chemical shifts of the amide protons with temperature and provide information about hydrogen bonding. While low Δ*δ*/Δ*T* values (–2.4 ± 0.5 ppb K^−1^) are attributed to both shielded and exposed protons and are therefore not meaningful, larger Δ*δ*/Δ*T* values always reflect initially shielded, HB-engaged amide protons that are exposed to the solvent during the unfolding of intramolecularly hydrogen-bonded structures or the dissociation of aggregates at elevated temperatures [13,14,76,77,78,79,80,81,82,83,84]. Considering that the concentration-independent IR and NMR spectra exclude the presence of self-assembled aggregates, the observed larger temperature coefficients further confirm the presence of intramolecularly folded conformations in peptides **1**–**4** (Table 2).

7. NOESY Spectroscopy

**Figure 4:**
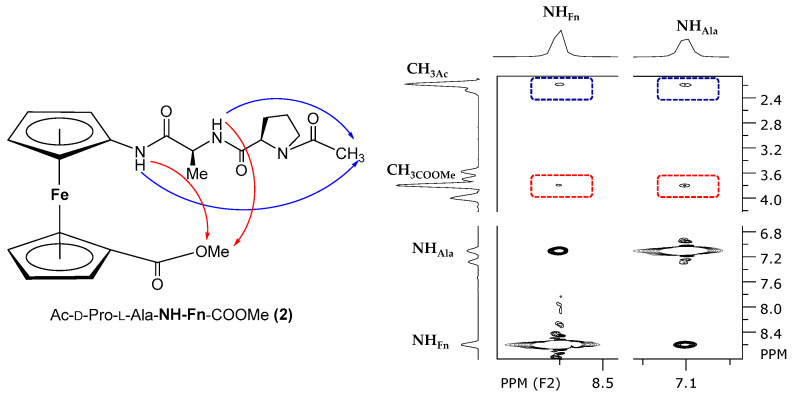
The NOE contacts in the spectra of conjugate **2** are depicted with arrows.

The IHB-mediated folding of peptides **1**–**4**, predicted by computational study, was further investigated by a NOE experiment. Considering the proposed conformational patterns **A**, **B**, and **C** (Figure 3), we focused on NOE cross peaks which support the close proximity of NH_Fn_/NH_Ala_ with the *N*-terminal Boc/Ac group and/or with the ester methyl group. As for the heterochiral peptides **1** and **2**, the observed NOE interactions clearly support the presence of IHB pattern **A** with two HBs. The NOE contact between the NH_Fn_ and acetamide methyl protons of peptide **2** indicates the presence of an intrastrand HB NH_Fn_···O=C_Ac_ corresponding to β-turns. In addition, the NOE contacts between NH_Fn_ and NH_Ala_ and the ester methyl group indicate the presence of an interstrand HB NH···O=C_COOMe_ (Figure 4). The targeted NOE interactions were not observed for homochiral peptides **3** and 4, which was expected given the 1D NMR data mentioned above, indicating that the homochirality of the peptide backbone does not contribute to the conformational stability.

8. CD Spectroscopy

Circular dichroism (CD) is an effective method of studying the conformational changes during aggregation, thermal or chemical unfolding, and ligand binding interactions [85,86]. After the coupling of ferrocene scaffolds with amino acids and peptides, the folding into turn- and β-sheet-like conformations stabilized by IHBs occurs, followed by the restricted free rotation of ferrocene rings. This leads to helical chirality of the ferrocene core, which causes the Cotton effects around 470 nm. The intensity of the Cotton effects is more pronounced when the folded conformations are more stable, and their positive or negative sign is related to the right- or left-handed helicity of the ferrocene core [87]. The results of the computational study together with the IR and NMR data indicate that the heterochirality of the peptide backbone contributes to conformational stability. Therefore, the almost two-fold stronger positive Cotton effects (M*_θ_*~4200–4800 deg cm^2^ dmol^−1^) observed for the heterochiral conjugates **1** and **2** were expected and are attributed to the presence of the conformational pattern **A**, stabilized by two hydrogen bonds. However, the decrease in and weakening of the hydrogen bonding in the homochiral peptides **3** and **4**, found to adopt single hydrogen-bonding pattern **B** or **C**, resulted in significantly weaker CD activity (Table 3).

#### 2.1.3. Biological Evaluation

Antitumor activity

**Figure 5:**
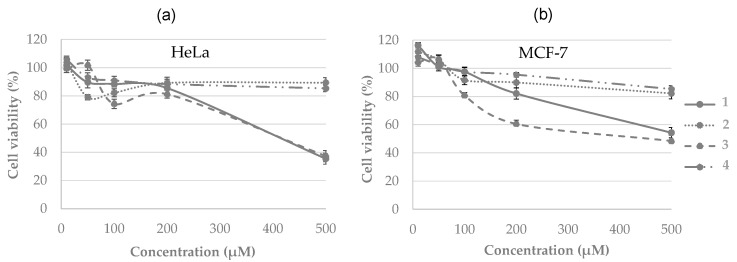
Cell viability of (**a**) HeLa and (**b**) MCF-7 cells treated with peptides **1**–**4** for 72 h in the range of concentration from 10 μM to 500 μM and assessed by the CellTiter 96^®^ AQueous One Solution Cell Proliferation Assay (Promega (Madison, WI, USA).

The conjugates **1**–**4** were evaluated for their ability to inhibit the growth of HeLa and MCF-7 carcinoma cells. Inhibitory activity was observed only at concentrations of 100 μM or higher. However, the Boc-protected peptides **1** and **3** were more efficient than Ac-protected peptides **2** and **4**, with the viability of cells treated with the highest concentration (500 μM) being from 35.4377% (HeLa) to 54.3296% (MCF-7) for **1** and from 37.2897% (HeLa) to 48.3693% (MCF-7) for **3** (Figure 5).

In vitro cytotoxicity results, quantified as the IC_50_ values (the concentration of a compound where the percentage of inhibition is equal to 50), reveal that homochiral Boc-peptide **3** has the strongest inhibitory effect on MCF-7 cells, while HeLa cells were found to be somewhat less sensitive. In addition, heterochiral Boc-peptide **1** exhibited inhibitory activity against HeLa cells. In the range of tested concentrations (10–500 μM), no 50% inhibition of cell growth was observed for compounds **1**, **2**, or **4** on MCF-7 cells, and for compounds **2** or **4** on HeLa cells (Table 4).

**Figure 6:**
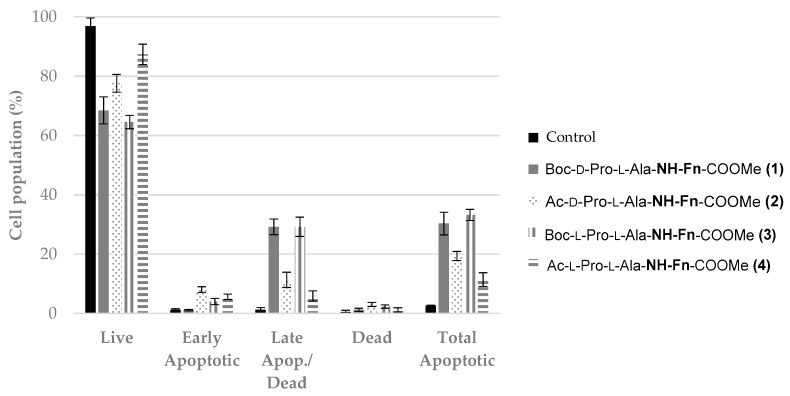
Cell death analysis of HeLa cells treated with the highest tested concentration of Boc- (**1** and **3**) and Ac-peptides (**2** and **4**) (500 µM) for 72 h.

Programmed cell death, or apoptosis, is a key biological process associated with many types of diseases, including autoimmune and inflammatory diseases and cancer [88]. Since peptidomimetics are known to induce apoptosis [89], HeLa cells were treated with conjugates **1**–**4**, followed by flow cytometry analysis. The results show that HeLa cells treated with Boc-peptide **3** (500 µM) had the highest percentage of the total apoptotic cells (33.19%), followed by its diasteremer **1** with a total apoptosis of 30.32% (Figure 6). The lowest percentage of total apoptotic cells was determined when HeLa cells were treated with Ac-peptide **4** (11.45%), which was shown to have the weakest effect on HeLa cells. We found that the biological activity of ferrocene peptides depends on their lipophilicity rather than their conformational properties. The ferrocene-containing peptides characterized with larger retention factors *(R*_f_) in non-polar eluents were shown to exert better antiproliferative activity [28]. Thus, the results described above regarding the increased antiproliferative activity of Boc-peptides **3** (*R*_f_ = 0.55) and **1** (*R*_f_ = 0.52) compared to the more polar Ac-peptides **2** (*R*_f_ = 0.33) and **4** (*R*_f_ = 0.35) are in accordance with our previous reports.

### 2.2. Unsymetrically Substituted Conjugates of Amino-Functionalized Ferrocene with Phe, Val, and Leu ***5**–**16*** [33]

The potential of antimicrobial peptides in combating pathogens is related to their structure and physicochemical properties, such as conformation, electrical charge, hydrophilicity, and hydrophobicity [90]. Therefore, we performed the synthesis, detailed conformational analysis, and biological evaluation of 12 conjugates of amino-functionalized ferrocene with Phe, Val, and Leu containing hydrophobic branched and bulky side chains [33].

#### 2.2.1. Computational Study

**Figure 7:**
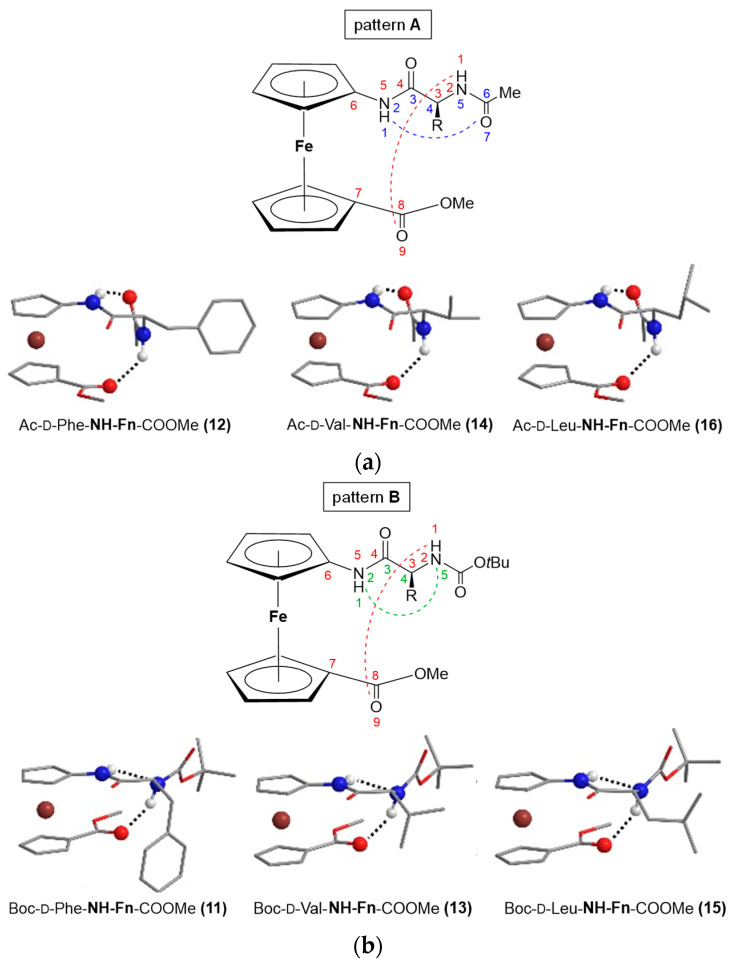
The DFT-predicted hydrogen-bonding patterns in (**a**) Ac- (**12**, **14**, and **16**) and (**b**) Boc-protected conjugates (**11**, **13,** and **15**). The 3D conformations are adopted from reference [33].

Since the same Boltzmann distribution and hydrogen bonding patterns are expected for each pair of enantiomers, the conformational analysis was performed only for the d-series of the prepared peptides. Hydrogen-bonding pattern **A**, which consists of two IHBs (intrastrand NH_Fn_···OC_Ac_ HB forming nine-membered ring and interstrand NH_AA_···OC_COOMe_ HB forming seven-membered ring), was adopted by Ac-protected conjugates **12**, **14**, and **16** (Figure 7a), while pattern **B**, which is based on nine- (interstrand NH_AA_···OC_COOMe_ HB) and five-membered hydrogen-bonded rings (intrastrand NH_Fn_···N_AA_ HB) was found to be inherent to the Boc-protected conjugates **11**, **13**, and **15** (Figure 7b).

#### 2.2.2. Spectroscopic Study

1. IR Spectroscopy

Since the enantiomers have identical chemical and physical scalar properties, the experimental IR and NMR data are given only for the d-series. The common feature of the amide region of the IR spectra of the tested peptides is the dominance of the blue-shifted signals of the non-bonded NH groups (~3420 cm^−1^), which is due to the steric hindrance of the bulky and branched side chains of Phe, Val, and Leu. In addition, the intensity ratios of the free and associated NH bands indicate that Ac-conjugates **12**, **14**, and **16** (*I*_Nhfree_/*I*_Nhassoc._~1.4/1) are more involved in hydrogen bonding than their counterparts **11**, **13**, and **15** (*I*_NHfree_/*I*_NHassoc._~3–4.4/1), which contain more bulky Boc-protecting groups (Table 5).

2. Concentration-dependent IR Spectroscopy

The unchanged ratio of free and associated NH bands at dilution from *c* = 5 × 10^−2^ M to 3 × 10^−3^ M excludes the possibility of intermolecular hydrogen bonding.

3. NMR Spectroscopy

In view of the fact that the hydrogen-bonded amide protons are downfield-shifted (*δ* > 7 ppm), it can be assumed that the NH_Fn_ of the tested peptides is involved in the HBs (Table 6). The higher chemical shifts of the NH_Fn_ in the Ac-peptides **12**, **14**, and **16** than in those of the Boc-protected counterparts **11**, **13**, and **15** indicate their participation in slightly stronger HBs. As with the Ac-peptides, the more pronounced downfield shifting of the NH_Fn_ of the Val- and Leu-conjugates **14** and **16** (*δ*~8.2 ppm) compared to the Phe-conjugate **12** (*δ*~7.7 ppm) is due to interference of the bulky benzyl side chain in the hydrogen bonding. The slightly upfield-shifted (*δ*~6.5 ppm) NH_AA_ of Ac-peptides **12**, **14**, and **16** most likely fluctuates between bonded and non-bonded states, while the upfield-shifted resonances of the NH_AA_ of Boc-peptides **11**, **13**, and **15** do not exclude their involvement in HBs, as urethane NH protons can be observed at high fields [91,92].

4. Concentration-dependent NMR Spectroscopy

The negligible changes (Δ*δ*~0.2–0.6 ppm) in the resonances of NH_Fn_ and NH_Ala_ observed upon dilution from 50 mM to 6 mM indicate their involvement in IHBs, as revealed by concentration-independent IR spectroscopy.

5. Solvent-dependent NMR Spectroscopy

The solvent dependance of the NH chemical shifts is a measure of the strength of hydrogen bonds. Thus, the pronounced changes in the chemical shifts of NH_Fn_ and NH_AA_ in Ac-peptides **12**, **14**, and **16** observed during titration with DMSO (Δ*d* = 1.44–1.87 ppm) indicate their involvement in weak IHBs (Table 6).

6. Temperature-dependent NMR Spectroscopy

The large temperature coefficients (Δ*δ*/Δ*T*) calculated for peptides **12**, **14**, and **16** are attributed to originally hydrogen-bonded NH protons that are exposed to the solvent at increased temperatures (Table 6).

7. CD Spectroscopy

In agreement with the results of the IR and NMR spectroscopy, which indicate a rather weak capacity of the tested peptides for hydrogen-bonding, only a weak CD activity was observed. In addition, the enantiomer pairs **5**/**11**, **6**/**12**, **7**/**13**, **8**/**14**, **9**/**15**, and **10**/**16** show Cotton effects with opposite signs and equal (or very similar) intensities (Table 7).

#### 2.2.3. Biological Evaluation

1. Antimicrobial activity

In terms of antibacterial activity, highly potent antibacterial ferrocene-peptide conjugates have been shown to be able to penetrate the bacterial membrane which leads to detachment of vital enzymes required for respiration and cell wall biosynthesis [93]. The ferrocene group has also been used to modify and enhance the activity of natural antioxidants [94,95]. The preliminary results for the antimicrobial activity of compounds **5**–**16** were obtained using the disk diffusion method. Only Ac-conjugates **12**, **14**, and **16** exhibited antimicrobial activity with inhibition zones of 7–19 mm against the bacteria *P. aeruginosa*, *B. subtilis*, and *S. aureus*. Among them, the Leu-containing conjugate **16** showed the strongest antimicrobial activity, especially against the bacterium *P. aeruginosa*. The Phe-containing peptide **12** had the same efficacy against the treated bacteria, while the Val-conjugate **14** showed the weakest inhibitory potential (Table 8).

2. Antioxidant activity

The antioxidant activity of conjugates **5**–**16** was tested using the 1,1-diphenyl-2-picrylhydrazyl (DPPH) and ferric-reducing antioxidant power (FRAP) methods. The results are expressed as mM Trolox for the FRAP method and as mM Trolox equivalent for the DPPH method. The results were compared using 0.5 mM Trolox as a reference substance (Table 9). The d-series conjugates **11**–**16** showed higher antioxidant activity as measured by the FRAP method, while l-peptides **6**, **8**, and **10** showed the lowest antioxidant activity as measured by both methods.

3. Hydrophobicity

In the development of peptide drugs, it is important to set an optimal ratio between hydrophilic and hydrophobic fractions at which the peptides pass through the membrane the most easily. The peptide must be hydrophobic enough to interact with the non-polar hydrocarbon chains of the fatty acids, but not lipophilic enough to get stuck in the membrane or be insoluble in water. The total hydrophobicity of the peptides **5**–**16** arises from the ferrocene and amino acid moieties and the *N*-terminal group (Boc/Ac), and was evaluated by measuring their retention times (*t_R_*). Besides the hydrophobicity, the peptide retention time is also related to the properties of the liquid chromatography system. Since the elution from the column with a hydrophobic stationary phase (C18) was carried out with a polar mobile phase (water/acetonitrile), the more hydrophobic peptides had longer retention times [96,97,98,99]. Considering the retention times listed in Table 10 and the results for the peptides’ antimicrobial activity shown in Table 8, the optimal ratio of hydrophilic/hydrophobic content is only given in d-series peptides with an Ac-protecting group (**12**, **14**, and **16**).

4. Chemical and proteolytic stability

**Figure 8:**
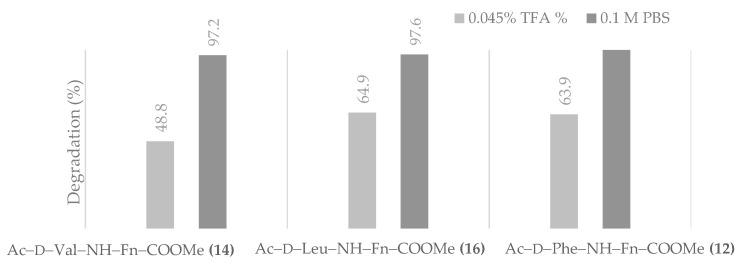
Percentage of degradation of Ac-peptides **12**, **14**, and **16** in 0.045% TFA and 0.1 M buffer after 24 h.

The two degradation assays, 0.1 M phosphate-buffered saline (PBS) buffer and 0.045% TFA, were used to test the chemical stability of Ac-conjugates **12**, **14**, and **16**, which have been shown to inhibit bacterial growth. As can be seen in Figure 8, the tested peptides were stable in the buffer at a neutral pH, whereas, in 0.045% TFA, significant degradation occurred within 24 h.

**Figure 9:**
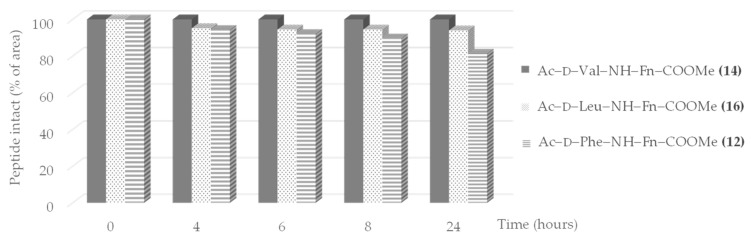
Proteolytic stability of ferrocene peptides **12**, **14**, and **16** during 24 h.

In terms of proteolytic stability, Val-peptide **14** proved to be the most stable to chymotrypsin degradation, while the Phe- (**12**) and Leu- (**16**) peptides were slightly more sensitive to proteolysis (peptides **12** and **16** showed a decrease in peak area of 18.7% and 6%, respectively) (Figure 9). Therefore, the high proteolytic stability of these ferrocene-d-amino acid conjugates represents a promising avenue for the discovery of new peptidomimetics with improved conformational and proteolytic stability and biological activity.

### 2.3. Symetrically Disubstituted Conjugates of Amino-Functionalized Ferrocene with Phe, Val and Leu ***17**–**22*** [34]

The pattern and strength of the hydrogen bonds in asymmetrically substituted conjugates of amino-functionalized ferrocene with Phe, Val, and Leu **5**–**16** were not suitable to achieve highly ordered chiral structures. To improve their conformational stability, we continued our research by preparing their higher, symmetrically disubstituted homologues Ac–AA–**NH**–**Fn**–**NH**–AA–Boc (AA = l- and d-Phe (**17**/**18**), l- and d-Val (**19**/**20**), and l- and d-Leu (**21**/**22**)). Considering the excellent conformational stability and chiral organization of the previously reported symmetrically disubstituted conjugate Ac–Ala–**NH–Fn–NH**–Ala–Boc [28], we were interested in investigating the influence of hydrophobic branched and bulky benzyl, isopropyl, and isobutyl side chains on both the hydrogen bonding between the peptide strands and the biological activity of conjugates **17–22**.

#### 2.3.1. Computational Study

**Figure 10:**
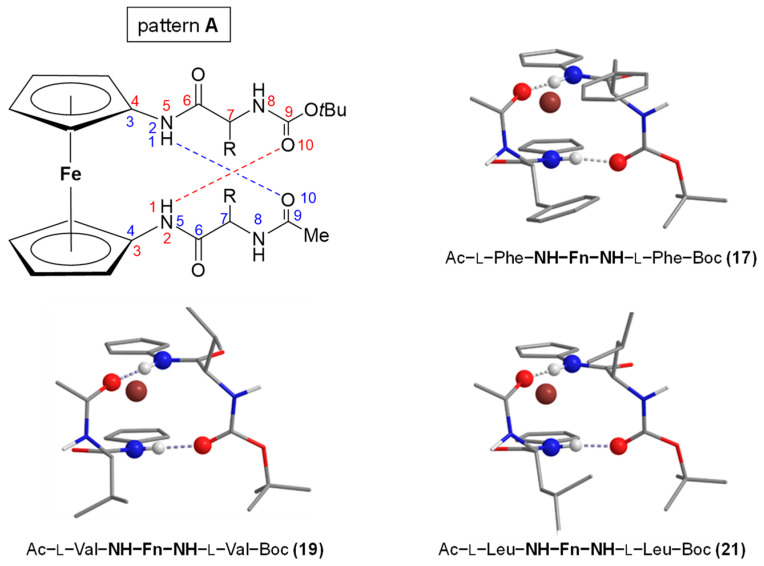
The DFT-predicted hydrogen-bonding patterns in conjugates **17**, **19**, and **21**. The 3D conformations are adopted from reference [34].

As mentioned above, the results of the conformational analysis are given only for the l-conjugates **17**, **19**, and **21**, since the same Boltzmann distribution is expected for their d-enantiomers **18**, **20**, and **22**. The most stable conformations were based on hydrogen-bonding pattern **A**, which consists of two 10-membered IHB rings, i.e., two β-turns connected by two hydrogen bonds between the peptide strands attached to the opposite cyclopentadienyl rings NH_Fn_···OC_Boc_ and NH_Fn_···OC_Ac_, respectively (Figure 10). The same motif was adopted by their Ala-analogue [28].

#### 2.3.2. Spectroscopic Study

1. IR Spectroscopy

The IR data of the asymmetrically monosubstituted conjugates consisting of a ferrocene unit and one amino acid strongly suggest the predominance of non-bonded states (Table 5) [33]. As can be seen in Table 11, their higher symmetrically disubstituted homologues **17**, **19**, and **21** are mainly involved in hydrogen bonding due to the presence of additional hydrogen-bond-donating and -accepting sites. In addition, the higher proportion of free NH bands in Phe-conjugate **17** (*I*_NHfree_/*I*_NHassoc_ = 0.7:1) indicates that benzyl side chains interfere with hydrogen bonding more than aliphatic side chains in Val-peptide **19** and Leu-peptide **21** (*I*_NHfree_/*I*_Nhassoc_ = 0.7:1).

2. Concentration-dependent IR Spectroscopy

The ratio of the intensities of free and bonded NH bands remained unchanged at a dilution from *c* = 5 × 10^−2^ M to 3 × 10^−3^ M, indicating that bonded NH groups are only involved intramolecularly.

3. NMR Spectroscopy

Since hydrogen bonding leads to the deshielding of the protons involved, the pronounced downfield shifts observed for NH_Fn_ (*δ* > 9 ppm) indicate their involvement in a strong HB, while the upfield-shifted NH_Ac/Boc_ is likely to be involved only in a weak HB or not be bonded at all (Table 12).

4. Concentration-dependent NMR Spectroscopy

Similar to the concentration-independent IR vibrations, the chemical shifts of NH_Fn_ were also found to be concentration-independent, with only minor changes (Δ*δ* < 0.16 ppm) when diluted from 50 mM to 6.25 mM, further confirming its participation in IHBs. However, a significant upfield shift of NH_Ac/Boc_ was observed (Δδ~0.6–1 ppm), which could indicate the presence of intermolecular aggregates.

5. Solvent-dependent NMR Spectroscopy

The involvement of NH_Fn_ in a strong IHBs due to its pronounced downfield shifts was further supported by a flat titration curve (chemical shift vs. percentage of DMSO), resulting from only negligible changes in chemical shifts (Δδ~0–0.16 ppm) (Table 12). As expected, the non- or weakly hydrogen-bonded and, therefore, exposed NH_Ac/Boc_ was solvated by DMSO (Δδ ≥ 1–1.7 ppm).

6. Temperature-dependent NMR Spectroscopy

The large temperature dependence (−5.71 to −8.71 ppb K^−1^) of the concentration- and DMSO-independent NH_Fn_ is attributed to its involvement in strong IHBs. The large temperature coefficients of concentration-dependent NH_Ac_ (−11.57 to −18.42 ppb K^−1^) are further evidence of its involvement in intramolecular HBs, while a small temperature dependence of NH_Boc_ reflects its non-involvement in HBs (Table 12).

7. NOESY Spectroscopy

**Figure 11:**
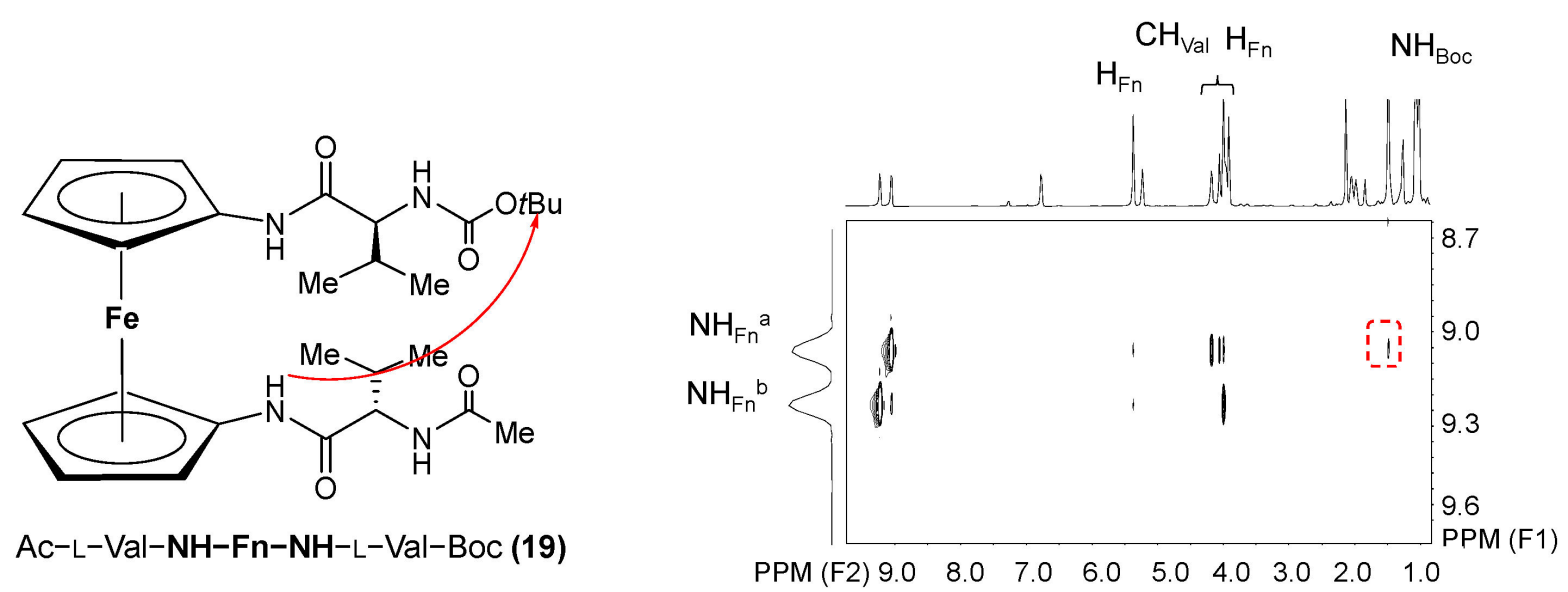
The NOE contacts in the spectra of conjugate **19** are depicted with arrows.

The NMR data on the involvement of NH_Fn_ in a strong IHB are consistent with the DFT-predicted hydrogen bonding pattern **A**, which is based on two interstrand IHBs: NH_Fn_···OC_Boc_ and NH_Fn_···OC_Ac_, i.e., two simultaneous β-turns (Figure 10). Therefore, the NOESY spectra of peptides **17**, **19**, and **21** were inspected for the presence of cross peaks between NH_Fn_ and protons of the terminal carbamate and acetamide groups. The observed interstrand NOE contacts between NH_Fn_ and the *tert*-butyl protons confirm the presence of interstrand IHB NH_Fn_···OC_Boc_ corresponding to β-turns (Figure 11).

8. CD Spectroscopy

The Ala-analogue of the tested compounds, Ac–Ala–NH–Fn–NH–Ala–Boc [28], was found to adopt the same conformational pattern **A** based on two simultaneous β-turns. The highly ordered chiral organization was reflected in a strong positive Cotton effect (*M*_θ_~23,000 deg cm^2^ dmol^−1^), which the intensity was not significantly decreased in the presence of DMSO (<30%), indicating an exceptional conformational stability. The Cotton effects shown in Table 13 are of opposite signs and very similar intensity for the enantiomeric pairs **17/18**, **19/20**, and **21/22**. While the Val- (**19/20**) and Leu-conjugates (**21/22**) show slightly improved conformational stability compared to the Ala-analogs, there is a noticeable decrease in the CD activity of the Phe-peptides **17/18** as a result of the interference of the bulky benzyl groups with the hydrogen bonding. The preservation of the CD activity in the presence of DMSO (75–90%) confirmed the previous IR-and NMR-indicated involvement of the tested peptides in IHB-mediated folding into stable β-turn structures.

#### 2.3.3. Biological Evaluation

1. Antitumor activity

**Figure 12:**
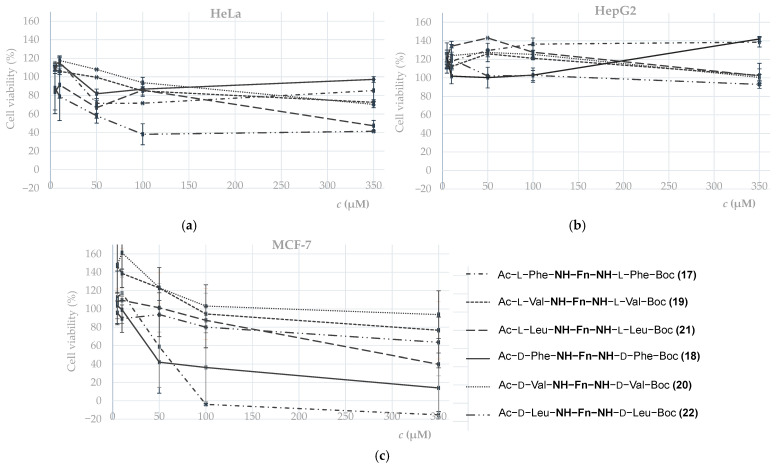
Dose–response curves for tested compounds **17**–**22** in (**a**) HeLa, (**b**) Hep G2, and (**c**) MCF-7 cells.

The NIH-NCI DTP protocol [100] was used to evaluate the antitumor activity of conjugates **17**–**22** on HeLa, HepG2, and MCF-7 cell lines. The common feature of the tested compounds is their inactivity against HepG2 cells and the strongest inhibition of cell growth in MCF-7 cells (Figure 12, Table 14). In addition, an inhibitory effect of more than 50% was achieved with l- and d-Leu peptides **21**/**22** in HeLa cells, and l- and d-Phe peptides **17**/**18** in MCF-7 cells, within the tested range. A negligible inhibitory effect was observed for l- and d-Val peptides **19**/**20**. Besides the cell line treated, the antitumor activity of ferrocene peptides also depends on the lipophilicity. Compared to the more polar Val- (**19/20** (*R*_f_ ~ 0.58)) and Leu- (**21/22** (*R*_f_ ~ 0.45)) peptides, Phe-conjugates **17/18**, with larger retention factors in less polar solvents (*R*_f_ ~ 0.83), showed the highest cytotoxic effect. Their IC_50_ values of 53.1 ± 23 μM and 32.7 ± 6.89 μM are lower than those for the reference drug cisplatin (97.86 μM) (Table 14).

**Figure 13:**
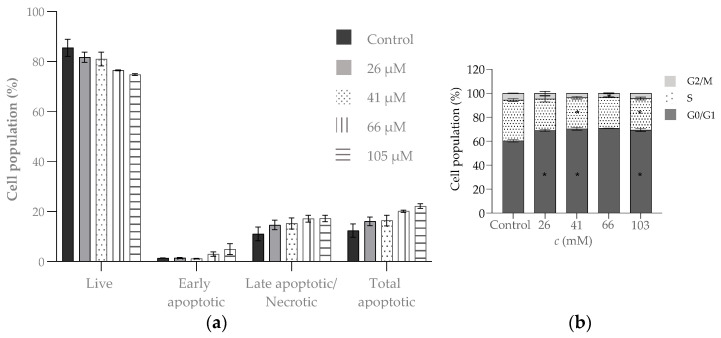
(**a**) The effect of peptidomimetic **22** on apoptosis of HeLa cells, (**b**) cell cycle profiles for control HeLa cells and cells treated with four different concentrations of compound **22** for 24 h. Cell cycle distribution was calculated as the percentage of cells in the G0/G1, S, and G2/M phase. Data were calculated as mean ± SEM. Statistical significance was considered if * *p* < 0.05.

The possible induction of apoptosis in HeLa cells by the action of d-Leu-conjugate **22** was investigated. As shown in Figure 13a, no significant difference was observed between the control and treated cells. However, the higher percentage of total apoptotic cells observed in the treatment with peptide **22** makes this conjugate a potent inducer of apoptosis in HeLa cells. In addition, the role of conjugate **22** in the cell cycle progression of HeLa cells was examined (Figure 13b). A significant increase in the number of cells in the G0/G1 phase was observed compared to control cells, followed by a significant decrease in the cell population in the S phase. These results suggest that peptide **22** inhibits the proliferation of HeLa cells, as it can induce cell cycle arrest in the G0/G1 phase.

2. Antioxidant activity

The symmetrically disubstituted conjugates **17**–**22** showed only moderate antioxidant activity in the range of 0.1 mM Trolox equivalent as assessed by DPPH and in the range of 0.52–0.63 mM Trolox as assessed by FRAP (Table 15). Looking at the results of the antioxidant activity of their lower homologues **6**, **8**, **10**, **12**, **14**, and **16** (Table 9), the introduction of an additional hydrophobic amino acid did not contribute to the improvement of the antioxidant activity.

## 3. Peptide Derivatives of Aminoferrocene—Origin of Chiroptical Activity

An intrinsic property of the bioconjugates of hetero-anullarly functionalized ferrocenes and various l-, d-, and β-amino acids is a helically chiral arrangement of the ferrocene moiety. In these derivatives, a prerequisite for the formation of helically chiral conformations is the presence of chiral centers in the peptide chains, which are connected by interchain HBs that restrict the free rotation of the ferrocene rings [9,28,29,56]. Depending on the chirality of the amino acids used, an opposite helicity of the metallocene core can be generated. In general, l-amino acids favour the *P*-helicity of the ferrocene moiety and vice versa. This helically chiral arrangement can be easily detected by recording the CD spectra in the visible region, where *P*-helicity causes positive Cotton effects near the apsorption maximum of the ferrocene chromophore (*λ* ~ 470 nm), while *M*-conformers induce negative CD signals. The intensity of these signals ranges from ±10^3^ deg cm^2^ dmol^−1^ to ±7 × 10^5^ deg cm^2^ dmol^−1^ and presumably depends on the stability of the folded conformations and, thus, on the degree of asymmetry in the vicinity of the metallocene chromophore.

In contrast to the derivatives described above, the chirality and CD activity of ferrocenes (aminoferrocene or ferrocenecarboxylic acid) substituted with only one peptide chain have been insufficiently investigated. The main reason for this is that these derivatives are usually present in solution as a mixture of rapidly changing conformations that are stabilized by weak if any non-covalent interactions. The ensemble of these interconverting conformations shows only weak CD signals, which were thought to originate from the central chirality of the individual amino acids. As mentioned above, an inherently achiral ferrocene chromophore must be placed in an ordered asymmetric environment to exhibit pronounced CD activity, and when considering these derivatives, asymmetric perturbation can be generated by the handedness of the dominant conformations in a hydogen-bonded peptide suquence. To date, there are only a few examples of amino acid derivatives of ferrocenecarboxylic acid in which ordered structural elements that could cause optical activity in the apsorption region of the ferrocene chromophore have been detected. Kraatz and co-workers [102] found a β-turn structure in the crystalline state of ferrocenecarboxylic acid substituted with a tripeptide chain (–l–Pro–l–Pro–l–Phe–COOBzl), but did not study its conformation in solution in detail nor did they record its CD spectrum. By introducing a heterochiral peptide chain into the ferrocenecarboxylic acid, Hirao [103] prepared the enantiomeric derivatives Fn–CO–l–Ala–d–Pro–NH–4–Py and Fn–CO–d–Ala–l–Pro–NH–4–Py, which favour a β-turn-like structure with the opposite sense of handedness, both in solution and in the solid state. This chiral environment leads to mirror-image signals in the visible region of their CD spectra. The first example of an experimental and theoretical investigation of the origin of the chiroptical activity of 1-ferrocenyl amino acids was recently given by Zhong [104]. He found that conjugation of a single chiral amino acid forces the carboxyl-functionalized ferrocene into a fixed chirality supported by three types of intramolecular interactions, including CH⋯π interactions, van der Waals interactions, and hydrogen bonding. He also found that the sign and the intensity of the CD signal at *λ* ~ 450 nm is determined by the orientation of the carbonyl group directly bonded to the ferrocene, i.e., the angle of rotation between it and the cyclopentadienyl ring.

Over the last 10 years, our group has been working intensively on the chirooptical properties of peptide derivatives of aminoferrocene and their potential application as CD sensors. The impetus for this was the realisation that even simple dipeptide derivatives of aminoferrocene such as *t*BuO-CO–AA_2_–AA_1_–NH-Fn [27,105] (AA_1_ = Gly, l-Ala, d-Ala, l-Val; AA_2_ = Gly, l-Ala, l-Val, l-Pro, d-Pro) form ordered structural elements: β-turn- and γ-turn-like structures, supported by intrachain HBs. Moreover, opposite signs of Cotton effects were observed in the CD spectra of the enantiomeric dipeptides depending on the chirality of the incorporated amino acids and, thus, the handedness of the turn-like structures. A detailed spectroscopic and DFT investigation led us to the conclusion that these CD signals undoubtedly originate from the transfer of chiral information from a folded peptide sequence to a ferrocene chromophore and that right-handed turns favored by l-amino acids give rise to negative CD signals and vice versa.

### 3.1. C-Terminal Ferrocene-Capped Tripeptides ***23**–**30*** [35]

Previous observations prompted us to investigate the use of ferrocene as a CD probe for the detection of screw sense preferences of short peptide sequences, for which we synthesized all eight stereoisomers of Boc–Pro–Pro–Ala–NH–Fn (**23**–**30**) [35]. This investigation was inspired by early work by Toniolo [106] and his *p*-bromobenzamido chromophore and Clayden’s dibenzazepinylurea [107], which were used as *N*-terminally covalently bound CD probes for determination and quantification of the screw sense preferences of peptide sequences. We opted for tripeptide sequences containing a diproline segment in addition to alanine because (i) the absence of NH groups in cyclic amino acids restricts the accessible conformational space, (ii) these derivatives can form α-turns or other helical structures induced by the sterically restricted diproline segment in addition to β-turns. Combined experimental and theoretical studies were performed to establish the clear link between the chiroptical properties of ferrocene and the local conformation of the peptide sequence. All derivatives were prepared by stepwise HOBt/EDC-mediated coupling technique in the solution phase, and conformational analysis was performed by spectroscopy (IR, NMR and CD), X-ray structural analysis and theoretical calculations. Since half of the target compounds are in an enantiomeric relationship and have the same scalar properties, the experimental data are described for only one series.

In the amide region of the IR spectra of dilute solutions of derivatives **23**–**25**, strong intensity signals attributable to hydrogen-bonded NH groups were registered, accompanied by almost negligible intensity bands belonging to free NH groups. Since tripeptides **23**–**25** contain only two NH groups, we assumed that both are involved in IHBs, while this is not the case for tripeptide **26**, in whose IR spectrum the signal that can be assigned to free NH groups is slightly stronger. This was also confirmed by the NMR data shown in Table 16. In the NMR spectra of all derivatives, the NH_Fn_ and NH_Ala_ groups resonate in the low field, while the NH_Ala_ group of **26** is detected in a significantly higher field, leading to the conclusion that the former groups are involved in IHBs, while this is questionable for the latter. Titration with DMSO allowed us to investigate the strength of these intramolecular interactions, and as can be seen from Table 16, the NH_Ala_ groups of peptides **23**–**25** as well as NH_Fn_ of **26** are involved in the strongest IHBs, whereas NH_Fn_ of **23**, **25** and **26** are involved in somewhat weaker interactions, while the large perturbation of the chemical shift of NH_Ala_ of **26** indicates its solvent exposure. Moreover, the initial downshifted resonances of NH_Fn_ and NH_Ala_ protons in **24** and **25** showed no significant temperature dependence, which is characteristic of shielded hydrogen-bonded protons. Higher values of the temperature coefficients of the NH_Fn_ groups of **23** and **26** as well as the NH_Ala_ group of **26** indicate an initially shielded proton exposed upon temperature increase, whereas the proton of the NH_Ala_ group of derivative **26** is free of hydrogen bonding when its initial resonance is considered. 

**Figure 14:**
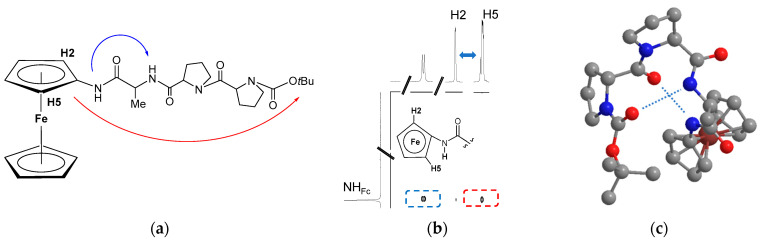
(**a**) Sequential NH-NH and long-range NOE interactions in the spectra of **2**3–26; (**b**) Anisochronicity of ferrocene H2 and H5 protons in the NOESY spectrum of **24** caused by fixed orientation of directly bound amide plane; (**c**) Consecutive β-turns in the crystal structure of **24**.

Analysis of the NOESY spectra provided us with additional evidence for hydrogen bond-induced folding of the peptide sequence in derivatives **23**–**26**, as NOE signals between the sequential NH groups as well as the long-range interactions between the ferrocene H2 and H5 hydrogens and the *N*-terminal *tert*-butyl group were observed in the spectra of all tripeptides (Figure 14a). Interestingly, we also observed that the cross-peaks of NH_Fn_ of **23** and **26** and the diastereotopic H2 and H5 atoms of ferrocene, whose chemical shifts are separated for 0.07 ppm for **23** and 0.03 ppm for **26**, are equally intense (Figure 14b). In contrast, in the NOESY spectra of derivative **24**, the diastereotopic protons were largely separated (0.6 ppm) and a strong signal was observed between NH_Fn_ and the shielded cyclopentadienyl proton; similar was found in the NOESY spectrum of **26**. These findings suggest that the same orientation of the amide bond directly attached to ferrocene predominates in the conformers of the later tripeptides, leading to a shielding of one of the diastereotopic protons by the influence of the carbonyl group and a fixed orientation of the shielded proton towards NH group. In the single-crystal X-ray structure of compound **24** no significant intermolecular interactions were observed, as both NH groups are involved in IHBs forming two consecutive β-turns, which can be classified as type II′ and type I (Figure 14c).

**Figure 15:**
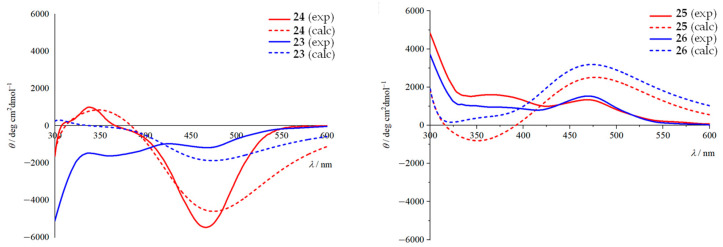
Measured (CH_2_Cl_2_, *c* = 1 × 10^−3^ mol·dm^−3^) and calculated (time-dependent density functional theory) CD curves of **23**–**26**.

Finally, we recorded and calculated the CD spectra of derivatives **23**–**30**, and as can be seen in Figure 15, they agree very well. The sign of the Cotton effect in the region of the metal-centred transitions is negative in the spectra of **23** and **24**, the spectra of **25** and **26** show a positive sign of the Cotton effect, while their enantiomeric **27**–**30** display mirror-image curves.

**Figure 16:**
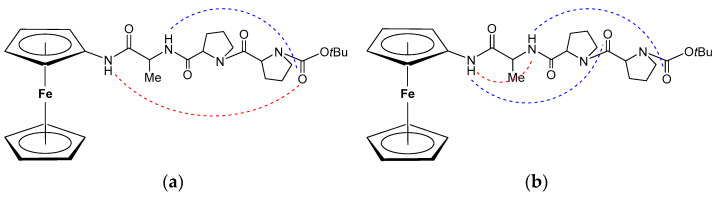
Hydrogen bonding pattern observed in the most stable conformer of (**a**) **23** and **26**; and (**b**) **24** and **25**.

In order to reconcile these experimental results with the molecular geometries, detailed calculation studies were carried out in the further course of the research. In the most stable conformations of compounds **23** and **26**, branched hydrogen bonds connect the urethane carbonyl and NH groups of aminoferrocene and Ala, forming 13- and 10-membered rings (Figure 16), while the predominant conformation of **24** and **25** contains two consecutive β-turns (NH_Fn_···O=C_Pro1_ and NH_Ala_···O=C_Boc_). With the exception of tripeptide **18**, other HB patterns were found in the calculated geometries of **17**, **19** and **20**, albeit in less energetically favorable conformations.

**Figure 17:**
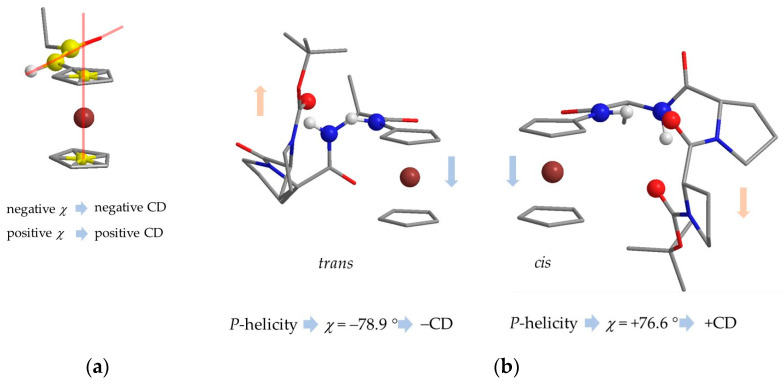
(**a**) *χ* angle defined by two centroids, nitrogen and amide carbon, an example in the figure has a negative *χ* angle; (**b**) The opposite orientation of the *P*-helical peptide chain to the ferrocene moiety (*cis*- or *trans*-) leads to opposite values of the *χ* angle and sign in the CD spectra.

At the beginning of our research, we postulated that the transfer of the *P*- or *M*-helicity of the turn or helix structure in the peptide segment to the ferrocene unit leads to CD signals with different signs in the visible region of the CD spectra. Unfortunately, after careful comparison of the calculated geomerties of **23**–**26** and their CD spectra, we could not correlate the helicity of the structures in the peptide sequence with the sign of the CD curves, e.g., a right-handed *P*-helix containing two consecutive β-turns found in the most stable conformers of **24** and **25** gives rise to the opposite signs of the measured and calculated CD signals at 470 nm. Interestingly, however, when analysing the data, we found that the observed turns not only cause a folding of the peptide sequence in the *P*- or *M*-direction, but also a deviation from the coplanarity of the plane of the cyclopentadienyl (Cp) ring and the directly bound amide plane. To describe this deviation, we used the dihedral angle *χ*, which we defined by four atoms, two centroids, one for each Cp ring, nitrogen and amide carbon (Figure 17a). The signs of the CD curves around 470 nm of all investigated geometries of peptides **23**–**26** showed a correlation with the sign of the *χ* angle, with positive values of *χ* leading to positive signals and vice versa. This discovery allowed us for the first time to identify the origin of the Cotton effects in the CD spectra of ferrocene peptides derived from aminoferrocene and their correlation with the geometry of the peptide sequence. But since ferrocene can adopt both a *cis*- and a *trans*-arrangement with respect to the peptide chain (Figure 17b), resulting in opposite values of the *χ* angle and thus opposite CD spectra, we were unfortunately unable to correlate the CD sign with the helicity of the peptide sequence. Nevertheless, this study provided guidelines for the design of ferrocene-based CD probes for determination of the screw-sense preferences of small helical peptide structures and the chirality of amino acids, which we focused on in the continuation of the research.

### 3.2. Transfer of Chiral Information in Aib Containing Aminoferrocene Peptides ***31**–**33*** [36]

As mentioned above, the major obstacle to correlation of the helicity of the peptide segment with the CD signal at around 470 nm is that ferrocene can adopt two orientations with respect to the peptide chain, namely *cis*- and *trans*-, resulting in opposite signals being registered despite equal helicity. To overcome this problem, we decided to introduce α-amino isobutyric acid (Aib), an achiral helicogenic amino acid known to promote 3_10_ helices [108], as a spacer between the ferrocene core and the chiral *N*-terminus [102]. We expected that the rubust 3_10_ helical geometries would force the ferrocene into a single orientation to the peptide chain, presumably *trans*-, and allow the transfer of chiral information stored at the *N*-terminus to the ferrocene core, enabling the correlation of CD signal and helicity. To develop a practical ferrocene CD probe, we prepared Boc–l–Ala–(Aib)*_n_*–NH–Fn (**31**, *n* = 1; **32**, *n* = 2; **33**, *n* = 3) and investigated their conformational and chiroptical properties by experimental and theoretical methods.

Continuing the research, we recorded IR spectra of dilute solutions of peptides **31**–**33** and found that as the number of amino acids increases, the proportion of associated NH groups also increases. Since NH_Fn_ as well as NH_Aib1_ of **32** and **33** and NH_Aib2_ of tetrapeptide **33** are registered in a low field in the NMR spectra (Table 17), it can be concluded that these groups are involved in hydrogen bonding. In contrast, the remaining central and the *N*-terminal NH groups of all derivatives were registered at about 6.5 and 5 ppm, from which it can be concluded that they are free of hydrgen bonding. DMSO titration (23%) and temperature-dependent NMR spectroscopy confirmed these assumptions, as the chemical shift of these groups changed significantly with the addition of DMSO but only slightly upon heating compared to the groups registered above 7 ppm. Moreover, observation of sequential dNN (*i*, *i* + 1) NOESY crosspeaks in the spectra of **31**–**33** strongly indicates their helical arrangement.

**Figure 18:**
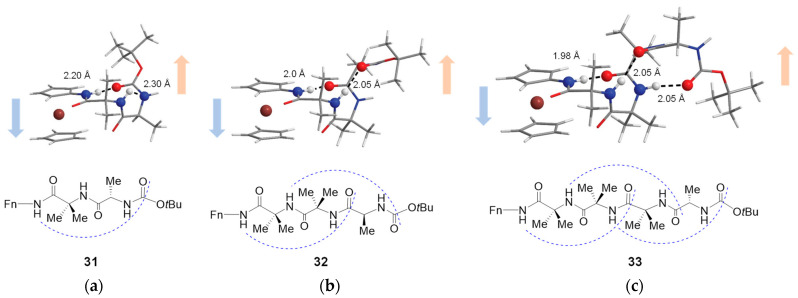
DFT optimized geometries of the most stable conformers of (**a**) **31**, (**b**) **32** and (**c**) **33** and hydrogen-bonding patterns containing 10-membered rings.

Considering the IR and NMR results, the primary structure of peptides **31**–**33** and the propensity of Aib to induce 3_10_ helical structures, we made the following assumptions: (i) dipeptide **31** is stabilized by a hydrogen bond between NH_Fn_ and CO_Boc_, (ii) two 10-membered rings involving NH groups at positions *i* + 3 and *i* + 4, and the carbonyl groups of the *i*-th and *i* + 1 residues stabilize tripeptide **32** and (iii) tetrapeptide **33** adopts a 3_10_-helix supported by successive hydrogen bonds involving *C*-terminal NH groups. These ordered elements alow the transfer of chiral information stored at the *N*-terminus to the ferrocene chromophore trough Aib spacer as all derivatives exhibit negative Cotton effects in the visible region, the intensity of which decreases with increasing number of amino acids contained. In the hope of finding a dominant *trans*-conformer that would allow a correlation between the sign of these signals and the helicity of the peptide sequence, the assembly of the most stable conformers of **31**–**33** (Figure 18) and their CD spectra were calculated. The lowest energy conformers of dipeptide **31** and tripeptide **32** contain one and two 10-membered rings, respectively, as shown by the experimental analysis with the ferrocene in *trans*-position to the peptide sequence. As in our previous studies, the negative *χ* angle value led to negative Cotton effects, which can be correlated with *P*-helicity in the case of the *trans*-conformers.

In addition to these conformers, further energetically accessible conformations were found for **31** and **32**. *P*-helical structures in which ferrocene is oriented in the *cis*-direction to the peptide sequence (separated from lowest energy trans conformer for 3.98 kJ mol ^−1^ for **31** and 4.64 kJ mol^−1^ for **32**), as well as additional geometries with different helicity and hydrogen bonding patterns between these two levels. In contrast to the inhomogeneous conformational space of **31** and **32**, tetrapeptide **33** exists mainly as a dominant *trans*-conformer in which the propagating *P*-helix causes a negative *χ* angle and CD signal. In the case of tetrapeptide **33**, the *cis*-conformer, in which the *P*-helix causes a positive divergence and thus a positive CD signal, is separated for 8.16 kJ mol^−1^.

To summarise, we have shown that the minimal sequence that exists primarily in a dominant, 3_10_ helical *trans*-geometry is (Aib)_3_-l-Ala. Apart from locking the conformation in *trans*-orientation, the robust achiral (Aib)_3_ segment transfers the chiral information from the *N*-terminus to ferrocene and allows correlation of the helicity of the peptide sequence with the sign of the CD signal (*P*-helicity induced by l-Ala → negative *χ* → negative CD). This study was an important step in the development of an aminoferrocene-based CD probe that could be used for the chiral discrimination of amino acid enantiomers and the determination of the helicity of peptides.

### 3.3. Central-to-Helical-to-Axial Chirality Transfer in Ac6c Aminoferrocene Peptides ***34**–**36*** [37]

Although in the previous study we confirmed the conformational homogeneity of the (Aib)_3_ sequence, which allowed the correlation of the CD signal with the helicity of peptide sequence (induced by chirality of the *N*-terminus), it is important to emphasize that the difficult copulation of multiple Aib residues reduces the potential of using (Aib)_3_–NH–Fn as a CD probe. Therefore, we decided to replace Aib with cyclic achiral Ac6c, i.e., to synthesize Ac6c–NH–Fn (**vii**) as a sensor and test its potential for determining the chirality of *N*-terminally bound amino acids via central-to-helical-to-axial chirality transfer [104]. In developing the sensor, we were guided by two assumptions: (*i*) the sterically demanding Ac6c will force the peptide substituent into a *trans*-conformation towards ferrocene in a similar way as (Aib)_3_, and (*ii*) the dominant conformation of Boc–AA–Ac6c–NH–Fn (AA = l-Ala, l-Val, l-Phe) dipeptides will be a right-handed β-turn geometry, i.e., the l-amino acid will induce a right-handed β-turn geometry, causing a negative value of the *χ* angle and thus a negative CD signal in the case of the *trans*-conformation of the substituent.

**Figure 19:**
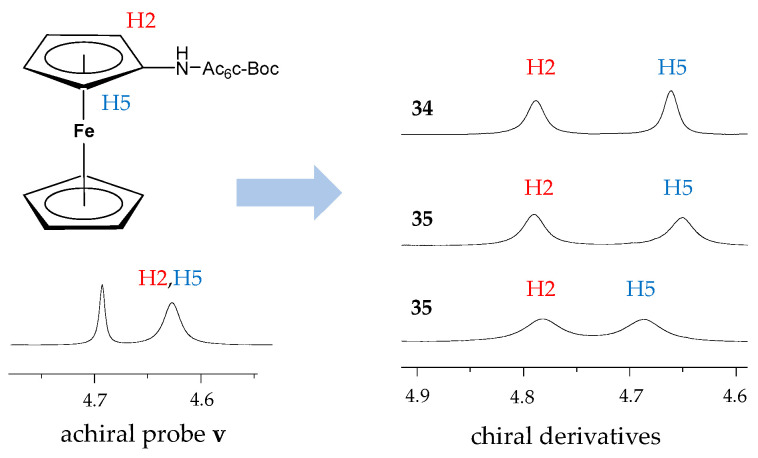
Isochronous ferrocene H2 and H5 protons of the probe and anisochronicity induced by *N*-therminal chiral induction in the spectra of **34**–**36**.

Based on the spectroscopic analysis and our previous studies of aminoferrocene-derived dipeptides, we hypothesised that **34**–**36** predominantly adopt the β-turn conformation in solution. Namely, in the NMR spectra of their dilute solutions, the chemical shifts of the NH_Fn_ group were registered in the low field (Δ*δ* ~ 8.40 ppm), while sequential signals characteristic of the β-turn geometry were found in the NOESY spectra. Since only one of the three NH groups in derivatives **34**–**36** is involved in β-turn, it is not surprising that we observed bands of both associated and free NH groups in their IR spectra. Moreover, the *N*-terminal chiral induction led to a separation of the signals of the diastereotopic ferrocene protons in ^1^H NMR spectra of **34**–**36**, in contrast to the spectra of the achiral sensor **v**, where they were registered as a single signal (Figure 19).

**Figure 20:**
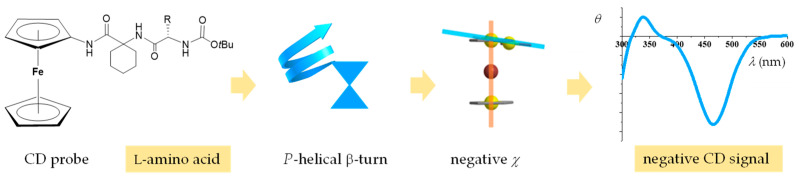
*P*-helical β-turn, induced by l-amino acid, gives rise to negative *χ* angle and CD signal.

DFT modeling and recording of CD spectra confirmed all our earlier assumptions (Figure 20). The predominant conformation of **34**–**36**, which is more than 75% occupied, is stabilized by a β-turn element supported by a hydrogen bond between the NH_Fn_ group and the urethane carbonyl. At the same time, the l-amino acids induce a right-handed conformation that is *trans*-oriented towards ferrocene due to the steric requirements of Ac6c, ultimately leading to negative values of the *χ* angle. As in our previous studies, these negative values cause negative Cotton effects in the measured and calculated CD spectra of derivatives **34**–**36**.

In summary, the conformational uniformity of the Ac6c–NH–Fn probe **v** bound to chiral amino acids and the central-helical-axial chirality transfer allowed us to correlate the chirality of the *N*-terminal amino acid with the sign of the CD signal in the ferrocene absorption region.

### 3.4. Dinuclear Ferrocene-Peptide Derivatives ***37*** and ***38*** [38]

**Figure 21:**
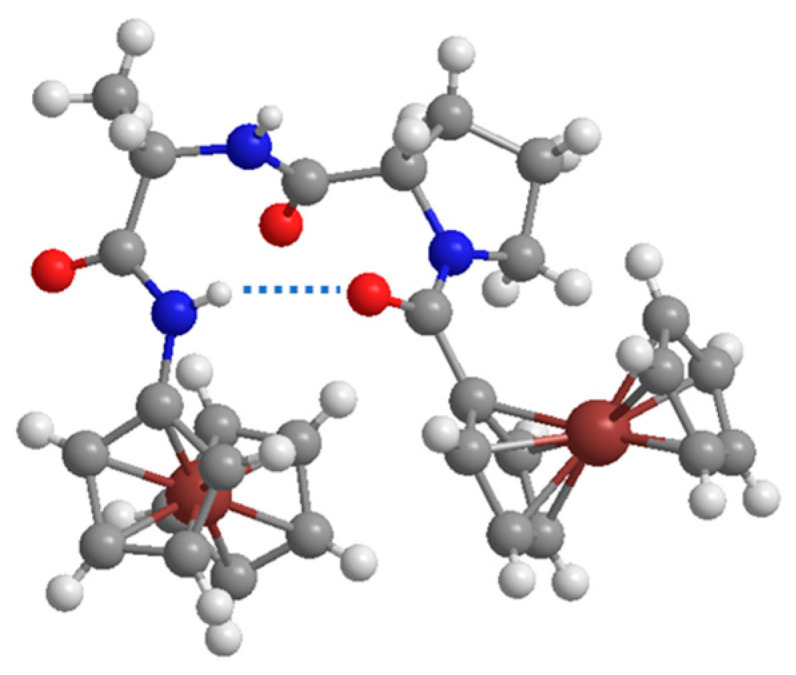
Type II β-turn in the crystal structure of **37**.

Inspired by previous studies [4,5] in which we have shown that the predominant conformation of homochiral ferrocene dipeptides is β-turn, which leads to strong signals in the visible region of the CD spectrum, we decided to investigate what effect the insertion of an additional ferrocene subunit at *N*-terminus has on the conformation and the CD signal. The enantiomeric Fn–CO–l–Pro–l–Ala–NH–Fn (**37**) and Fn–CO–d–Pro–d–Ala–NH–Fn (**38**) were prepared using a HOBt/EDC-mediated coupling procedure and subjected to experimental and theoretical conformational analysis [100]. Since in the IR spectra of diluted solutions of **37** and **38** bands of free and associated NH groups have approximately the same intensity, while in the NMR spectra the *C*-terminal NH group resonates at 8.3 ppm and the NH_Ala_ group at 6.7 ppm, we assumed that the former is involved in intramolecular hydrogen bonding. The high temperature coefficient of this group (Δ*δ*/Δ*T* = –6.1 ppb K^–1^), indicating an initially shielded group that is exposed upon heating, and the small perturbation (Δ*δ* = 0.6 ppm) of the chemical shift during DMSO titration (25%) confirmed this assumption. In contrast, the upfield shifted NH_Ala_ group shows a low temperature dependence (Δ*δ*/Δ*T* = 2.1 ppb K^–1^) and a pronounce change in the chemical shift (Δ*δ* > 1 ppm) upon addition of 25% DMSO. The sequential dNN (*i*, *i* + 1) and dαN (*i*, *i* + 1) NOE cross-peaks registered in the spectra of **37** and **38** indicate a type I β-turn conformation [109] stabilized by hydrogen bonding between amino and carbonyl groups directly attached to the ferrocene moieties. As expected, we observed signals of equal intensity but opposite sign in the CD spectra of enantiomeric **31** and **32**. The l,l-derivative **37** shows a CD signal of the same sign (negative) but lower intensity than the mononuclear precursor Boc–Pro–Ala–NH–Fn [5], and vice versa. DFT modeling found several conformers, dominated by those containing β-turns. The most energetically favorable conformation of **37** and **38** contains a β-turn of type I as indicated by experimetal analysis, while the one with type II, which is also determined in the crystal structure of compounds **37** and **38** (Figure 21), is somewhat more unstable.

In summary, the dominant conformation of dinuclear ferrocene derivatives bridged by a homochiral dipeptide segment in solution and in the solid state, as in the case of aminoferrocene bearing dipeptide sequences, is a robust β-turn.

## 4. Oxalamide-Bridged Ferrocene 39 [39]

In continuation of our pioneer work on ferrocene-oxalamides [30], we have prepared dimer –(CO–Ala–NH–Fn–COOMe)_2_ **39** as the higher homologue of compound –(CO–NH–Fn–COOMe)_2_, with two Ala units inserted into oxalamide bridge [39].

### 4.1. Computational Study

**Figure 22:**
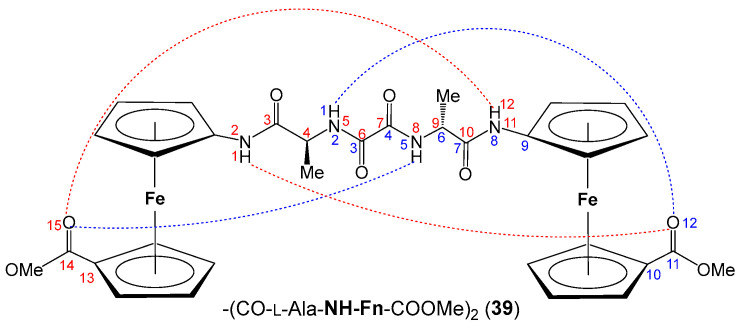
The DFT predicted hydrogen-bonding pattern in oxalamide **39**.

DFT study indicates the presence of two pairs of IHBs NH_Fn_···OC_COOM_e and NH_Ala_···OC_COOMe_ defining 15- and 12-membered rings, respectively. As can be seen in Figure 22, all NH groups are involved in IHBs.

### 4.2. Spectroscopic Study

1. IR Spectroscopy

Due to its insolubility in non-hydrogen bonding solvents CHCl_3_ and CH_2_Cl_2_, the IR spectra of oxalamide **39** were measured in tetrahydrofuran (THF) and toluene. Since no bands were observed in the region of the free NH bands (above 3400 cm^−1^), the NH groups are exclusively involved in intra- or/and intramolecular HBs (Table 18). However, the solvation effect of a weakly hydrogen-bond-accepting THF should be taken into account.

2. Concentration-dependent IR Spectroscopy

When the THF and toluene solutions of oxalamide **39** were diluted, the intensities of the NH bands decreased, but no blue-shifted band of NH_free_ appeared, indicating the involvement of NH groups in IHBs.

3. NMR Spectroscopy

Since the amide protons accessible to the hydrogen bond acceptor are downfield shifted [110,111], the high values of the chemical shifts of NH_Fn_ and NH_Ala_ of conjugate **39** suggest their involvement in HBs in THF and toluene (Table 19).

4. Concentration-dependent NMR Spectroscopy

The intramolecular nature of the hydrogen bonds suggested by IR spectroscopy is further supported by the almost unchanged chemical shifts of NH during dilution of the THF and toluene solutions.

5. Solvent-dependent NMR Spectroscopy

The pronounced downfield shift of the NH_Fn_ of oxalamide **39** in the presence of DMSO (Δ*δ* = 0.9 ppm) is indicative of their involvement in a weaker IHBs, while the almost unperturbed and solvent-shielded NH_Ala_ (Δ*δ* = 0.15 ppm) indicate their involvement in a stronger IHBs (Table 19).

6. Temperature-dependent NMR Spectroscopy

The larger temperature dependencies of the NH protons of compound **39** in THF and toluene are related to the unfolding of the initially IHB-driven folded structures.

7. NOESY Spectroscopy

**Figure 23:**
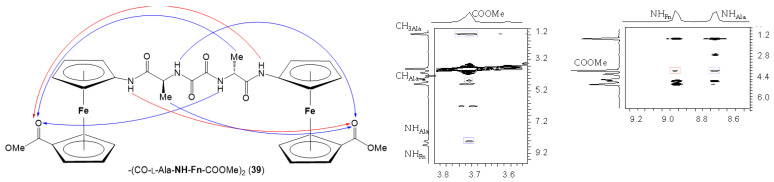
The NOE contacts in the spectra of oxalamide **39** are depicted with arrows.

Considering that both IR and NMR data strongly suggest the involvement of all NH groups in the IHBs, we analyzed their contacts in the NOESY spectrum. The interchain NOE contacts of the methyl ester protons with NH_Fn_, NH_Ala_ and CH_3Ala_ (Figure 23) provided additional evidence for a IHB pattern based on two pairs of hydrogen bonds: NH_Fn_···OC_COOM_e and NH_Ala_···OC_COOMe_ (Figure 22).

8. CD Spectroscopy

As expected, the oxalamide **39** which contains chiral Ala units and adopts IHB pattern shown in Figure 22, exhibited the positive Cotton effect (*M*_θ_ = 3422 deg cm^2^ dmol^−1^ in toluene) in the region of ferrocene-based transitions.

### 4.3. Gelation Properties

The lower homologue –(CO–NH–Fn–COOMe)_2_ [30] did not succeed in gelling a number of organic solvents. The gelling ability of its higher homologue **39** was only tested in toluene due to its low solubility. Although the longer Ala-Ala spacer in oxalamide **39** improves its flexibility, gelation of toluene was only possible with the aid of sonication.

### 4.4. Biological Evaluation

1. Antitumor activity

**Figure 24:**
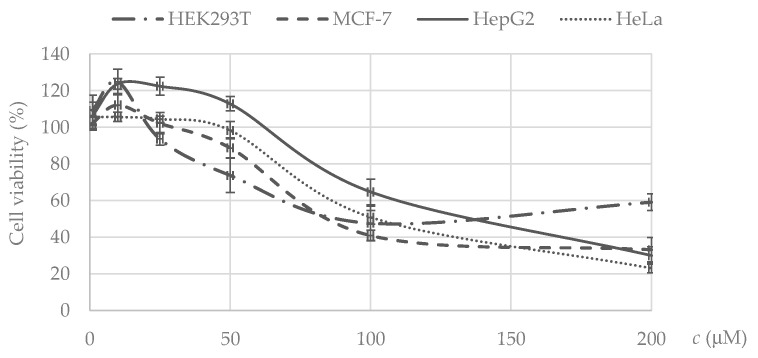
Dose response curves for oxalamide **39** in HEK293T, MCF-7, HepG2 and HeLa cells.

In general, the oxalamide derivatives have shown to be effective antitumor agents [112,113]. Therefore, the cytotoxic potential of ferrocene oxalamides **39** against three tumor cell lines (MCF-7, HeLa and HepG2) and one normal human cell line (HEK293T) was tested (Figure 24, Table 20). The compound **39** showed better cytotoxic activity in comparison to its lower homologue which was not only found to be inactive against HepG2 and HEK293T cells, but also had 2-fold higher IC_50_ value for HeLa cells.

## 5. Conclusions and Future Perspective

The aminoferrocene-peptide conjugates have not yet been investigated by other authors for the relationship between their conformational and biological properties, which made it impossible for us to evaluate our results in comparison with other relevant studies. To overcome the potential limitations and weaknesses of the applied methods for conformational evaluation of the goal peptides, they were used in combination with the aim of unambiguously confirming the results of each individual method. Moreover, the results of the combined theoretical and experimental approaches were in full agreement. The basic hypothesis about the most stable conformational pattern, i.e., the hydrogen-bonding pattern, was established by a DFT study. Then IR spectroscopy was performed to determine the proportion of hydrogen-bonded states, while NMR data not only provided information on the amide protons involved in the hydrogen bonds, but also indicated the strength of the hydrogen bonds. Finally, the CD activity of the tested peptides was in accordance with the IR and NMR results. To ensure an even more accurate defining of the conformational properties of ferrocene peptides, the described integrated DFT/spectroscopy approach should be extended with molecular dynamics simulations in our future work.

We have determined an efficient synthetic route to obtain the conjugates of the turn-inducing aminoferrocene scaffolds with amino acids and short peptides. In-depth conformational analysis based on DFT predictions corroborated with spectroscopic findings has revealed the three most important factors for the folding of ferrocene peptides into stable turn structures:

(i) The heterochirality of the peptide backbone contributes significantly to IHB-mediated folding into stable turn structures: the concentration-independent IR and concentration-, temperature- and solvent-independent NMR spectra of heterochiral conjugates Boc/Ac–d–Pro–l–Ala–NH–Fn–COOMe (**1/2**) indicate their participation in a strong intrastrand NH_Fn_···O=C_Boc/Ac_ HB corresponding to β-turn, and in an additional interstrand NH_Ala_···O=C_COOMe_ HB forming a 9-membered ring. Their homochiral counterparts Boc/Ac–l–Pro–l–Ala–NH–Fn–COOMe (**3/4**) are involved in more flexible structures.
(ii) Steric hindrance by the amino acid side chain significantly weakens conformational stability: the amide region of the IR spectra of the peptides Boc–AA–NH–Fn–COOMe (AA = d-Phe (**11**), d-Val (**13**), d-Leu (**15**)), Ac–AA–NH–Fn–COOMe (AA = d-Phe (**12**), d-Val (**14**), d-Leu (**16**)) is characterized by the dominance of the blue-shifted signals of the non-bonded NH groups, which is due to steric hindrance by the bulky and branched side chains of Phe, Val and Leu. In addition, the intensity ratios of the free and associated NH bands indicate that the bulky Boc-protecting groups in peptides **11**, **13** and **15** hinder the involvement in hydrogen bonding more than the Ac-group in **12**, **14** and **16**. Accordingly, their chemical shifts showed a pronounced dependence on temperature and solvent, and only weak Cotton effects are observed in the CD spectra.
(iii) The symmetrically disubstituted conjugates are involved in stronger IHBs and therefore adopt more stable conformations compared to asymmetrically monosubstituted peptides: while the asymmetrically monosubstituted conjugates **11**–**16** were found to be mostly non-involved in HBs, their higher symmetrically disubstituted homologues Ac–AA–NH–Fn–NH–AA–Boc (AA = l- and d-Phe (**17**/**18**), l- and d-Val (**19**/**20**), l- and d-Leu (**21**/**22**)) with additional hydrogen-bond-donor and acceptor sites were mainly involved in HBs. The most stable conformations were based on two 10-membered IHB rings, i.e., two β-turns connected by two hydrogen bonds between the peptide strands attached to the opposite cyclopentadienyl rings NH_Fn_···OC_Boc_ and NH_Fn_···OC_Ac_, respectively. The chemical shifts of the NH protons involved in HBs were temperature- and DMSO-independent. Moreover, the Cotton effects in their CD spectra were more than 20-fold stronger than those of peptides **11**–**16**.

In the last ten years, the antitumor activity of ferrocene peptidomimetics has only been tested in our group. We have shown that the ferrocene template and hydrogen-bonding pattern had no decisive influence on their activity, which was evaluated as low or moderate. The only structural feature associated with a slightly improved antitumor effect is lipophilicity.

In view of the above results, we will focus our future work on the heterochiral analogs of the peptides described here. To improve the lipophilicity, their depsipeptide analogs, in which an amide group is replaced by an ester group, will be synthesized and their conformational and biological properties will be evaluated. We believe that the results obtained will allow us to create a small library of ferrocene peptidomimetics with a defined structure-activity relationship.

Electronic circular dichroism is a powerful technique for determining the absolute configuration of chiral compounds, optical purity, secondary structure and folding properties of peptides and proteins, and various interactions. Unfortunately, the chirality of many compounds cannot be investigated directly with CD because the analytes do not contain strong chromophores and/or the absorption maximum lies at low wavelengths in the absorption range of other absorbing chromophores and solvents, the specific rotation is small and high sample concentrations are required [114]. These problems can be overcome by using achiral probes that undergo asymmetric induction upon binding to a chiral molecule, resulting in a strong characteristic CD output [115]. In our recent studies, we have shown that achiral aminoferrocene can undergo asymmetric perturbation upon binding to a peptide sequence and exhibit CD signals in the visible range, far from the absorption maxima of common solvents and other chromophores. In addition, we were able to describe the origin of these CD signals for the first time, which forms the basis for the use of ferrocene chromophores for the structural elucidation of chiral molecules.

## Figures and Tables

**Table 1:** The IR data of conjugates **1**–**4** (*c* = 5 × 10^−2^ M in CH_2_Cl_2_).

Compound	NH_free_ (ῠ_max_/cm^−1^)	NH_assoc._ (ῠ_max_/cm^−1^)	*I*_NHfree_/*I*_NHassoc._
Boc–d–Pro–l–Ala–NH–Fn–COOMe (**1**)	3418	3325	1:2.4
Ac–d–Pro–l–Ala–NH–Fn–COOMe (**2**)	3424	3303	1:2.3
Boc–l–Pro–l–Ala–NH–Fn–COOMe (**3**)	3418	3310	1:1.4
Ac–l–Pro–l–Ala–NH–Fn–COOMe (**4**)	3420	3309	1:1.6

**Table 2:** ^1^H NMR data for peptides **1**–**4** (*c* = 1 × 10^−3^ M in CDCl_3_).

Compound	NH_Fn_	NH_Ala_	NH_Fn_	NH_Ala_	NH_Fn_	NH_Ala_
	*δ* (ppm)		Δ*δ* (ppm)		Δ*δ*/Δ*T* (ppb K^–1^)	
Boc–d–Pro–l–Ala–NH–Fn–COOMe (**1**)	8.37	7.24	0.45	0.94	−7.42	−6.14
Ac–d–Pro–l–Ala–NH–Fn–COOMe (**2**)	8.58	7.08	0.3	1.46	−5.42	−3.85
Boc–l–Pro–l–Ala–NH–Fn–COOMe (**3**)	8.15	6.85	0.9	1.12	−8.57	−0.08
Ac–l–Pro–l–Ala–NH–Fn–COOMe (**4**)	7.99	7.22	0.98	0.83	−6.14	−4.14

**Table 3:** CD data for conjugates **1**–**4** (*c* = 1 × 10^−3^ M in CH_2_Cl_2_).

Compound	*M*_θ_ (deg cm^2^ dmol^−1^)
Boc–d–Pro–l–Ala–NH–Fn–COOMe (**1**)	+4230
Ac–d–Pro–l–Ala–NH–Fn–COOMe (**2**)	+4766
Boc–l–Pro–l–Ala–NH–Fn–COOMe (**3**)	−2481
Ac–l–Pro–l–Ala–NH–Fn–COOMe (**4**)	+205

**Table 4:** IC_50_ values calculated from dose–response curves of cell viability on HeLa and MCF7 cells.

	IC_50_ (μM)	
Compound	HeLa Cells	MCF-7 Cells
Boc–d–Pro–l–Ala–NH–Fn–COOMe (**1**)	436.1959	n.d. ^1^
Ac–d–Pro–l–Ala–NH–Fn–COOMe (**2**)	n.d. ^1^	n.d. ^1^
Boc–l–Pro–l–Ala–NH–Fn–COOMe (**3**)	370.3969	270.6925
Ac–l–Pro–l–Ala–NH–Fn–COOMe (**4**)	n.d. ^1^	n.d. ^1^

^1^ n.d. = not detected.

**Table 5:** The IR data of conjugates **11**–**16** (*c* = 5 × 10^−2^ M in CH_2_Cl_2_).

Compound	NH_free_ (ῠ_max_/cm^−1^)	NH_assoc._ (ῠ_max_/cm^−1^)	*I*_NHfree_/*I*_NHassoc._
Boc–d–Phe–NH–Fn–COOMe (**11**)	3412	3336	4.4:1
Ac–d–Phe–NH–Fn–COOMe (**12**)	3417	3295	1.5:1
Boc–d–Val–NH–Fn–COOMe (**13**)	3421	3328	3:1
Ac–d–Val–NH–Fn–COOMe (**14**)	3420	3289	1.4:1
Boc–d–Leu–NH–Fn–COOMe (**15**)	3422	3333	2.8:1
Ac–d–Leu–NH–Fn–COOMe (**16**)	3423	3289	1.4:1

**Table 6:** ^1^H NMR data for peptides **11**–**16** (*c* = 1 × 10^−3^ M in CDCl_3_).

Compound	NH_Fn_	NH_AA_	NH_Fn_	NH_AA_	NH_Fn_	NH_AA_
	*δ* (ppm)		Δ*δ* (ppm)		Δ*δ*/Δ*T* (ppb K^–1^)	
Boc–d–Phe–NH–Fn–COOMe (**11**)	7.32	5.15				
Ac–d–Phe–NH–Fn–COOMe (**12**)	7.73	6.44	1.72	1.54	−14.14	−9.14
Boc–d–Val–NH–Fn–COOMe (**13**)	7.38	5.15				
Ac–d–Val–NH–Fn–COOMe (**14**)	8.19	6.46	1.19	0.9	−16.86	−7.86
Boc–d–Leu–NH–Fn–COOMe (**15**)	7.62	5.03				
Ac–d–Leu–NH–Fn–COOMe (**16**)	8.15	6.38	1.23	1.37	−15.43	−11.28

**Table 7:** CD data for conjugates **6**–**16** (*c* = 1 × 10^−3^ M in CH_2_Cl_2_).

Compound	*M*_θ_ (deg cm^2^ dmol^−1^)
Boc–l–Phe–NH–Fn–COOMe (**5**)	679
Boc–d–Phe–NH–Fn–COOMe (**11**)	−679
Ac–l–Phe–NH–Fn–COOMe (**6**)	672
Ac–d–Phe–NH–Fn–COOMe (**12**)	−667
Boc–l–Val–NH–Fn–COOMe (**7**)	1228
Boc–d–Val–NH–Fn–COOMe (**13**)	−1216
Ac–l–Val–NH–Fn–COOMe (**8**)	675
Ac–d–Val–NH–Fn–COOMe (**14**)	−687
Boc–l–Leu–NH–Fn–COOMe (**9**)	960
Boc–d–Leu–NH–Fn–COOMe (**15**)	−957
Ac–l–Leu–NH–Fn–COOMe (**10**)	456
Ac–d–Leu–NH–Fn–COOMe (**16**)	−469

**Table 8:** Growth inhibition zones of the tested microorganisms.

Compound	Test Microorganisms	Molarity of Solutions for Disk Diffusion Method (mM)	Inhibition Zone (mm)
Ac–d–Phe–NH–Fn–COOMe (**12**)	*S. aureus* *B. subtilis* *P. aeruginosa*	224	14 ± 1
			14 ± 1
			14 ± 1
Ac–d–Val–NH–Fn–COOMe (**14**)	*S. aureus* *B. subtilis* *P. aeruginosa*	251	n.d. ^1^
			7 ± 1
			n.d. ^1^
Ac–d–Leu–NH–Fn–COOMe (**16**)	*S. aureus* *B. subtilis* *P. aeruginosa*	243	16 ± 1
			16 ± 1
			19 ± 2
Kanamycin	*S. aureus* *B. subtilis* *P. aeruginosa*	50 µg disk	25 ± 0
			25 ± 0
			28 ± 1

^1^ n.d. = not detected.

**Table 9:** Antioxidative activity of ferrocene compounds.

Compound (1 mM)	DPPH (% Inhibition)	DPPH (mM Trolox Equivalent)	FRAP (mM Trolox)
Boc–l–Phe–NH–Fn–COOMe (**5**)	5.50 ± 3.87	0.067	1.62 ± 0.001
Boc–d–Phe–NH–Fn–COOMe (**11**)	3.99 ± 0.48	0.051	2.00 ± 0.001
Ac–l–Phe–NH–Fn–COOMe (**6**)	3.65 ± 2.26	0.046	1.09 ± 0.030
Ac–d–Phe–NH–Fn–COOMe (**12**)	5.25 ± 2.26	0.067	2.22 ± 0.006
Boc–l–Val–NH–Fn–COOMe (**7**)	11.75 ± 2.10	0.150	2.16 ± 0.013
Boc–d–Val–NH–Fn–COOMe (**13**)	7.76 ± 0.65	0.099	2.17 ± 0.015
Ac–l–Val–NH–Fn–COOMe (**8**)	4.57 ± 0.64	0.058	0.58 ± 0.009
Ac–d–Val–NH–Fn–COOMe (**14**)	10.62 ± 1.13	0.135	2.29 ± 0.003
Boc–l–Leu–NH–Fn–COOMe (**9**)	16.55 ± 1.77	0.211	2.27 ± 0.006
Boc–d–Leu–NH–Fn–COOMe (**15**)	8.79 ± 2.10	0.112	2.21 ± 0.003
Ac–l–Leu–NH–Fn–COOMe (**10**)	3.19 ± 1.29	0.041	0.72 ± 0.004
Ac–d–Leu–NH–Fn–COOMe (**16**)	6.96 ± 0.81	0.089	1.77 ± 0.001
Standard (0.5 mM Trolox)	39.27 ± 8.40	0.500	

**Table 10:** List of the peptides used in this study.

Compound	*t_R_* (min)	% ACN
Boc–l–Phe–NH–Fn–COOMe (**5**)	8.3	87.9
Boc–d–Phe–NH–Fn–COOMe (**11**)	8.3	87.9
Ac–l–Phe–NH–Fn–COOMe (**6**)	3.6	68.0
Ac–d–Phe–NH–Fn–COOMe (**12**)	3.6	68.0
Boc–l–Val–NH–Fn–COOMe (**7**)	7.1	82.6
Boc–d–Val–NH–Fn–COOMe (**13**)	7.1	82.6
Ac–l–Val–NH–Fn–COOMe (**8**)	2.6	63.7
Ac–D–Val–NH–Fn–COOMe (**14**)	2.6	63.7
Boc–l–Leu–NH–Fn–COOMe (**9**)	8.3	87.5
Boc–d–Leu–NH–Fn–COOMe (**15**)	8.3	87.5
Ac–l–Leu–NH–Fn–COOMe (**10**)	3.3	66.6
Ac–d–Leu–NH–Fn–COOMe (**16**)	3.3	66.6

**Table 11:** The IR data of conjugates **17, 19**, and **21** (*c* = 5 × 10^−2^ M in CH_2_Cl_2_).

Compound	NH_free_ (ῠ_max_/cm^−1^)	NH_assoc._ (ῠ_max_/cm^−1^)	*I*_NHfree_/*I*_NHassoc._
Ac–l–Phe–NH–Fn–NH–l–Phe–Boc (**17**)	3430	3302, 3266, 3217	0.7:1
Ac–l–Val–NH–Fn–NH–l–Val–Boc (**19**)	3434	3305, 3249	0.5:1
Ac–l–Leu–NH–Fn–NH–l–Leu–Boc (**21**)	3434	3301, 3253	0.5:1

**Table 12:** ^1^H NMR data for peptides **17**, **19**, and **21** (*c* = 1 × 10^−3^ M in CDCl_3_).

Compound	NH_Fn_^a^	NH_Fn_^b^	NH_Ac_	NH_Boc_
	*δ* (ppm)			
Ac–l–Phe–NH^a^–Fn–NH^b^–l–Phe–Boc (**17**)	9.15	9.21	7.24	5.32
Ac–l–Val–NH^a^–Fn–NH^b^–l–Val–Boc (**19**)	9.02	9.29	6.74	5.29
Ac–l–Leu–NH^a^–Fn–NH^b^–l–Leu–Boc (**21**)	9.13	9.36	7.28	5.14
	NH_Fn_^a^	NH_Fn_^b^	NH_Ac_	NH_Boc_
	Δ*δ* (ppm)			
Ac–l–Phe–NH^a^–Fn–NH^b^–l–Phe–Boc (**17**)	0.16	0.07	1.44	1.77
Ac–l–Val–NH^a^–Fn–NH^b^–l–Val–Boc (**19**)	0.11	0.15	1.29	1.52
Ac–l–Leu–NH^a^–Fn–NH^b^–l–Leu–Boc (**21**)	0	0.05	0.96	1.9
	NH_Fn_^a^	NH_Fn_^b^	NH_Ac_	NH_Boc_
	Δ*δ*/Δ*T* (ppb K^–1^)			
Ac–l–Phe–NH^a^–Fn–NH^b^–l–Phe–Boc (**17**)	−8.71	−8.0	−18.42	−2.29
Ac–l–Val–NH^a^–Fn–NH^b^–l–Val–Boc (**19**)	−7.14	−5.71	−11.57	−1.42
Ac–l–Leu–NH^a^–Fn–NH^b^–l–Leu–Boc (**21**)	−6.57	−6.57	−17.71	−1.57

**Table 13:** CD data for conjugates **17**–**22** (*c* = 1 × 10^−3^ M in CH_2_Cl_2_).

Compound	*M*_θ_ (deg cm^2^ dmol^−1^)
Ac–l–Phe–NH–Fn–NH–l–Phe–Boc (**17**)	19.542
Ac–d–Phe–NH–Fn–NH–d–Phe–Boc (**18**)	−20.197
Ac–l–Val–NH–Fn–NH–l–Val–Boc (**19**)	25.725
Ac–d–Val–NH–Fn–NH–d–Val–Boc (**20**)	−26.442
Ac–l–Leu–NH–Fn–NH–l–Leu–Boc (**21**)	25.357
Ac–d–Leu–NH–Fn–NH–d–Leu–Boc (**22**)	−28.407

**Table 14:** IC_50_ values calculated from dose–response curves on HeLa, HepG2, and MCF-7 cells.

	IC_50_ (μM)		
Compound	HeLa	HepG2	MCF-7
Ac–l–Phe–NH–Fn–NH–l–Phe–Boc (**17**)	>350	>350	53.1 ± 23
Ac–d–Phe–NH–Fn–NH–d–Phe–Boc (**18**)	>350	>350	32.7 ± 6.89
Ac–l–Val–NH–Fn–NH–l–Val–Boc (**19**)	>350	>350	>350
Ac–d–Val–NH–Fn–NH–d–Val–Boc (**20**)	>350	>350	>350
Ac–l–Leu–NH–Fn–NH–l–Leu–Boc (**21**)	331 ± 39	>350	261 ± 97
Ac–d–Leu–NH–Fn–NH–d–Leu–Boc (**22**)	80.8 ± 15	>350	>350
Cisplatin	46.14 ^a^	15.9 ^b^	97.86 ^a^

^a^ Adapted from reference [56]. ^b^ Adapted from ref [101].

**Table 15:** Antioxidative activity of ferrocene conjugates **17**–**22**.

Compound (*c* = 1 mM)	FRAP (mM Trolox)	DPPH (% Inhibition of 0.1 mM Trolox)
Ac–l–Phe–NH–Fn–NH–l–Phe–Boc (**17**)	0.63 ± 0.21	113.77 ± 4.02
Ac–d–Phe–NH–Fn–NH–d–Phe–Boc (**18**)	0.58 ± 0.20	116.80 ± 18.19
Ac–l–Val–NH–Fn–NH–l–Val–Boc (**19**)	0.52 ± 0.06	102.62 ± 20.66
Ac–d–Val–NH–Fn–NH–d–Val–Boc (**20**)	0.62 ± 0.15	88.68 ± 24.52
Ac–l–Leu–NH–Fn–NH–l–Leu–Boc (**21**)	0.56 ± 0.17	81.98 ± 7.04
Ac–d–Leu–NH–Fn–NH–d–Leu–Boc (**22**)	0.60 ± 0.10	122.75 ± 2.17
Trolox 0.1 mM	-	100 ± 0.0

**Table 16:** ^1^H NMR data for peptides **2**3–**26** (*c* = 1 × 10^−3^ M in CDCl_3_).

Compound	NH_Fn_	NH_Ala_	NH_Fn_	NH_Ala_	NH_Fn_	NH_Ala_
	*δ* (ppm)		Δ*δ* (ppm)		Δ*δ*/Δ*T* (ppb K^–1^)	
Boc–l–Pro–l–Pro–l–Ala–NH–Fc (**23**)	8.26	7.67	0.07	0.07	−4	−4.8
Boc–d–Pro–l–Pro–l–Ala–NH–Fn (**24**)	8.26	7.11	0.36	0.03	−2.4	−0.8
Boc–l–Pro–d–Pro–l–Ala–NH–Fn (**25**)	8.04	7.52	0.55	0.8	−2.8	−2.8
Boc–d–Pro–d–Pro–l–Ala–NH–Fn (**26**)	8.58	6.50	0.34	1.96	−6.4	0.8

**Table 17:** ^1^H NMR data for peptides **31**–**33** (*c* = 1 × 10^−3^ M in CDCl_3_).

Compound	NH_Fn_	NH_Aib1_	NH_Aib2_	NH_Aib3_	NH_Ala_
	*δ* (ppm)				
Boc–l–Ala–Aib–NH–Fn (**31**)	8.38	6.45			5.11
Boc–l–Ala–Aib–Aib–NH–Fn (**32**)	8.33	7.04	6.55		5.10
Boc–l–Ala–Aib–Aib–Aib–NH–Fn (**33**)	8.51	7.46	7.39	6.81	5.50
	NH_Fn_	NH_Aib1_	NH_Aib2_	NH_Aib3_	NH_Ala_
	Δ*δ* (ppm)				
Boc–l–Ala–Aib–NH–Fn (**31**)	0.1	0.8			1.6
Boc–l–Ala–Aib–Aib–NH–Fn (**32**)	0.2	0.7	0.6		1.6
Boc–l–Ala–Aib–Aib–Aib–NH–Fn (**33**)	0.1	0.4	0.1	1.0	1.5
	NH_Fn_	NH_Aib1_	NH_Aib2_	NH_Aib3_	NH_Ala_
	Δ*δ*/Δ*T* (ppb K^–1^)				
Boc–l–Ala–Aib–NH–Fn (**31**)	−3.2	−0.1			−2.1
Boc–l–Ala–Aib–Aib–NH–Fn (**32**)	−2.2	−4.8	−0.6		−1.9
Boc–l–Ala–Aib–Aib–Aib–NH–Fn (**33**)	−2.8	−5.8	−6.3	−0.8	−0.8

**Table 18:** The IR data of conjugates **39**.

Compound		NH_free_ (ῠ_max_/cm^−1^)	NH_assoc._ (ῠ_max_/cm^−1^)
–(CO–Ala–NH–Fn–COOMe)_2_ (**39**)	THF	/	3382, 3294, 3252
	toluene	/	3305, 3268

**Table 19:** ^1^H NMR data for oxalamide **39**.

Compound		NH_Fn_	NH_Ala_	NH_Fn_	NH_AA_	NH_Fn_	NH_AA_
		*δ* (ppm)		Δ*δ* (ppm)		Δ*δ*/Δ*T* (ppb K^–1^)	
–(CO–Ala–NH–Fn–COOMe)_2_ (**39**)	THF	8.67	8.44	0.9	0.15	−7.7	−5
	toluene	7.90	8.36	/	/	−7	−12

**Table 20:** IC_50_ values for oxalamides **39** and its lower homologue calculated for HeLa, MCF7, HepG2 and HEK293Tcells.

	IC_50_ (μM)	
	–(CO–Ala–NH–Fn–COOMe)_2_ (39)	–(CO–NH–Fn–COOMe)_2_
HEK293T	94.88	n.d. ^a,b^
MCF-7	104.45	/
HepG2	140.34	n.d. ^a,b^
HeLa	110.20	252.79

^a^ n.d. = not detected; ^b^ Adapted from ref. [30].

## References

[1] Kealy T.J., Pauson P.L. (1951). New Type of Organo-Iron Compound. Nature.

[2] Miller S.A., Tebboth J.A., Tremaine J.F. (1952). Dyclopentadienyliron. J. Chem. Soc..

[3] https://www.chemistryworld.com/podcasts/ferrocene/6134.article.

[4] Patra M., Gasser G. (2017). The medicinal chemistry of ferrocene and its derivatives. Nat. Rev. Chem..

[5] Astruc D. (2017). Why is Ferrocene so Exceptional?. Eur. J. Inorg. Chem..

[6] Wang R., Chen H., Yan W., Zheng M., Zhang T., Zhang Y. (2020). Ferrocene-containing hybrids as potential anticancer agents: Current developments, mechanisms of action and structure-activity relationships. Eur. J. Med. Chem..

[7] Rauf U., Shabir G., Bukhari S., Albericio F., Saeed A. (2023). Contemporary Developments in Ferrocene Chemistry: Physical, Chemical, Biological and Industrial Aspects. Molecules.

[8] Ornelas C., Astruc D. (2023). Ferrocene-Based Drugs, Delivery Nanomaterials and Fenton Mechanism: State of the Art, Recent Developments and Prospects. Pharmaceutics.

[9] Čakić Semenčić M., Barišić L. (2017). Ferrocene Bioconjugates. Croat. Chem. Acta.

[10] Schlotter K., Boeckler F., Hübner H., Gmeiner P. (2005). Fancy Bioisosteres:  Metallocene-Derived G-Protein-Coupled Receptor Ligands with Subnanomolar Binding Affinity and Novel Selectivity Profiles. J. Med. Chem..

[11] Jaouen G., Vessières A., Top S. (2015). Ferrocifen type anti cancer drugs. Chem. Soc. Rev..

[12] Chavain N., Vezin H., Dive D., Touati N., Paul J.F., Buisine E., Biot C. (2008). Investigation of the redox behavior of ferroquine, a new antimalarial. Mol. Pharm..

[13] Sethi S., Kumar Das P., Behera N. (2016). The chemistry of aminoferrocene, Fe{(η^5^-C_5_H_4_NH_2_)(η^5^-Cp)}: Synthesis, reactivity and applications. J. Organomet. Chem..

[14] Kumaravel S., Balamurugan T.S.T., Jia S.-H., Lin H.-Y., Huang S.-T. (2020). Ratiometric electrochemical molecular switch for sensing hypochlorous acid: Applicable in food analysis and real-time in-situ monitoring. Anal. Chim. Acta.

[15] Wang W., Lu J., Hao L., Yang H., Song X., Si F. (2021). Electrochemical detection of alkaline phosphatase activity through enzyme-catalyzed reaction using aminoferrocene as an electroactive probe. Anal. Bioanal. Chem..

[16] Kumaravel S., Jian S.-E., Huang S.-T., Huang C.-H., Hong W.-Z. (2022). Convenient and ultrasensitive detection of live Salmonella using ratiometric electrochemical molecular substrates. Anal. Chim. Acta.

[17] Wang Y., Hong Y., Wang M., Zhu Y. (2022). Multifunctional Nanolabels Based on Polydopamine Nanospheres for Sensitive Alpha Fetoprotein Electrochemical Detection. ACS Appl. Nano Mater..

[18] Sariga, Varghese A. (2023). The Renaissance of Ferrocene-Based Electrocatalysts: Properties, Synthesis Strategies, and Applications. Top. Curr. Chem..

[19] Hagen H., Marzenell P., Jentzsch E., Wenz F., Veldwijk M.R., Mokhir A. (2012). Aminoferrocene-based prodrugs activated by reactive oxygen species. J. Med. Chem..

[20] Peiro Cadahia J., Previtali V., Troelsen N.S., Clausen M.H. (2019). Prodrug strategies for targeted therapy triggered by reactive oxygen species. MedChemComm.

[21] Daum S., Toms J., Reshetnikov V., Gizem Özkan H., Hampel F., Maschauer S., Hakimioun A., Beierlein F., Sellner L., Schmitt M. (2019). Identification of Boronic Acid Derivatives as an Active Form of N-Alkylaminoferrocene-Based Anticancer Prodrugs and Their Radiolabeling with 18F. Bioconjug. Chem..

[22] Gizem Özkan H., Toms J., Maschauer S., Prante O., Mokhir A. (2021). Aminoferrocene-Based Anticancer Prodrugs Labelled with Cyanine Dyes for in vivo Imaging. Eur. J. Inorg. Chem..

[23] Xu H.G., Schikora M., Sisa M., Daum S., Klemt I., Janko C., Alexiou C., Bila G., Bilyy R., Gong W. (2021). An Endoplasmic Reticulum Specific Pro-amplifier of Reactive Oxygen Species in Cancer Cells. Angew. Chem. Int. Ed..

[24] Gizem Özkan H., Thakor V., Xu H.G., Bila G., Bilyy R., Bida D., Böttcher M., Mougiakakos D., Tietze R., Mokhir A. (2022). Anticancer Aminoferrocene Derivatives Inducing Production of Mitochondrial Reactive Oxygen Species. Chem. Eur. J..

[25] Xu J., Tan J., Song C., Zhang G., Hu X., Liu S. (2023). Self-Immolative Amphiphilic Poly(ferrocenes) for Synergistic Amplification of Oxidative Stress in Tumor Therapy. Angew. Chem. Int. Ed..

[26] Wilde M., Arzur D., Baratte B., Lefebvre D., Robert T., Roisnel T., Le Jossic-Corcos C., Bach S., Corcos L., Erb W. (2020). Regorafenib analogues and their ferrocenic counterparts: Synthesis and biological evaluation. New J. Chem..

[27] Čakić Semenčić M., Kodrin I., Barišić L., Nuskol M., Meden A. (2017). Synthesis and Conformational Study of Monosubstituted Aminoferrocene-Based Peptides Bearing Homo- and Heterochiral Pro-Ala Sequences. Eur. J. Inorg. Chem..

[28] Kovačević M., Kodrin I., Cetina M., Kmetič I., Murati T., Čakić Semenčić M., Roca S., Barišić L. (2015). The conjugates of ferrocene-1,1′-diamine and amino acids. A novel synthetic approach and conformational analysis. Dalton Trans..

[29] Kovačević M., Kodrin I., Roca S., Molčanov K., Shen Y., Adhikari B., Kraatz H.-B., Barišić L. (2017). Helically Chiral Peptides That Contain Ferrocene-1,1′-diamine Scaffolds as a Turn Inducer. Chem.—Eur. J..

[30] Kovač V., Radošević K., Bebek A., Makarević J., Štefanić Z., Barišić L., Žinić M., Rapić V. (2017). The first oxalamide-bridged ferrocene: Facile synthesis, preliminary conformational analysis and biological evaluation. Appl. Organometal. Chem..

[31] Tomasini C., Castellucci N. (2013). Peptides and peptidomimetics that behave as low molecular weight gelators. Chem. Soc. Rev..

[32] Kovačević M., Čakić Semenčić M., Radošević K., Molčanov K., Roca S., Šimunović L., Kodrin I., Barišić L. (2021). Conformational Preferences and Antiproliferative Activity of Peptidomimetics Containing Methyl 1′-Aminoferrocene-1-carboxylate and Turn-Forming Homo- and Heterochiral Pro-Ala Motifs. Int. J. Mol. Sci..

[33] Kovačević M., Čakić Semenčić M., Kodrin I., Roca S., Perica J., Mrvčić J., Stanzer D., Molčanov K., Milašinović V., Brkljačić L. (2023). Biological Evaluation and Conformational Preferences of Ferrocene Dipeptides with Hydrophobic Amino Acids. Inorganics.

[34] Kovačević M., Markulin D., Zelenika M., Marjanović M., Lovrić M., Polančec D., Ivančić M., Mrvčić J., Molčanov K., Milašinović V. (2022). Hydrogen Bonding Drives Helical Chirality via 10-Membered Rings in Dipeptide Conjugates of Ferrocene-1,1′-Diamine. Int. J. Mol. Sci..

[35] Nuskol M., Šutalo P., Đaković M., Kovačević M., Kodrin I., Čakić Semenčić M. (2021). Testing the Potential of the Ferrocene Chromophore as a Circular Dichroism Probe for the Assignment of the Screw-Sense Preference of Tripeptides. Organometallics.

[36] Nuskol M., Šutalo P., Kodrin I., Čakić Semenčić M. (2022). Sensing of the Induced Helical Chirality by the Chiroptical Response of the Ferrocene Chromophore. Eur. J. Inorg. Chem..

[37] Nuskol M., Šutalo P., Kovačević M., Kodrin I., Čakić Semenčić M. (2023). Central-to-Helical-to-Axial Chirality Transfer in Chiroptical Sensing with Ferrocene Chromophore. Inorganics.

[38] Nuskol M., Studen B., Meden A., Kodrin I., Čakić Semenčić M. (2019). Tight turn in dipeptide bridged ferrocenes: Synthesis, X-ray structural, theoretical and spectroscopic studies. Polyhedron.

[39] Kovač V., Kodrin I., Radošević K., Molčanov K., Adhikari B., Kraatz H.-B., Barišić L. (2022). Oxalamide-Bridged Ferrocenes: Conformational and Gelation Properties and In Vitro Antitumor Activity. Organometallics.

[40] Barišić L., Rapić V., Kovač V. (2002). Ferrocene Compounds. XXIX. Efficient Syntheses of 1′-Aminoferrocene-1-carboxylic Acid Derivatives. Croat. Chem. Acta.

[41] Barišić L., Čakić M., Mahmoud K.A., Liu Y.-N., Kraatz H.-B., Pritzkow H., Kirin S.I., Metzler-Nolte N., Rapić V. (2006). Helically Chiral Ferrocene Peptides Containing 1′-Aminoferrocene-1-Carboxylic Acid Subunits as Turn Inducers. Chem. Eur. J..

[42] Wang X., Ni D., Liu Y., Lu S. (2021). Rational Design of Peptide-Based Inhibitors Disrupting Protein-Protein Interactions. Front Chem..

[43] Seychell B.C., Beck T. (2021). Molecular basis for protein-protein interactions. Beilstein J. Org. Chem..

[44] Lu H., Zhou Q., He J., Jiang Z., Peng C., Tong R., Shi J. (2020). Recent advances in the development of protein-protein interactions modulators: Mechanisms and clinical trials. Signal Transduct. Target Ther..

[45] Ovek D., Abali Z., Zeylan M.E., Keskin O., Gursoy A., Tuncbag N. (2022). Artificial intelligence based methods for hot spot prediction. Curr. Opin. Struct. Biol..

[46] Hoang H.N., Hill T.A., Ruiz-Gómez G., Diness F., Mason J.M., Wu C., Abbenante G., Shepherd N.E., Fairlie D.P. (2019). Twists or turns: Stabilising alpha vs. beta turns in tetrapeptides. Chem. Sci..

[47] Kim C., Jung J., Tung T.T., Park S.B. (2016). β-Turn mimetic-based stabilizers of protein-protein interactions for the study of the non-canonical roles of leucyl-tRNA synthetase. Chem. Sci..

[48] Laxio Arenas J., Kaffy J., Ongeri S. (2019). Peptides and peptidomimetics as inhibitors of protein-protein interactions involving β-sheet secondary structures. Curr. Opin. Chem. Biol..

[49] Vagner J., Qu H., Hruby V.J. (2008). Peptidomimetics, a synthetic tool of drug discovery. Curr. Opin. Chem. Biol..

[50] Lenci E., Trabocchi A. (2020). Peptidomimetic toolbox for drug discovery. Chem. Soc. Rev..

[51] Huan Y., Kong Q., Mou H., Yi H. (2020). Antimicrobial Peptides: Classification, Design, Application and Research Progress in Multiple Fields. Front. Microbiol..

[52] Kundu R. (2020). Cationic Amphiphilic Peptides: Synthetic Antimicrobial Agents Inspired by Nature. ChemMedChem.

[53] Liscano Y., Oñate-Garzón J., Delgado J.P. (2020). Peptides with Dual Antimicrobial–Anticancer Activity: Strategies to Overcome Peptide Limitations and Rational Design of Anticancer Peptides. Molecules.

[54] Giannis A., Rübsam F., Testa B., Meyer U.A. (1997). Peptidomimetics in Drug Design. Advances in Drug Research.

[55] Nair R.V., Baravkar S.B., Ingole T.S., Sanjayan G.J. (2014). Synthetic turn mimetics and hairpin nucleators. Chem. Commun..

[56] Barišić L., Dropučić M., Rapić V., Pritzkow H., Kirin S.I., Metzler-Nolte N. (2004). The first oligopeptide derivative of 1′-aminoferrocene-1-carboxylic acid shows helical chirality with antiparallel strands. Chem. Commun..

[57] Moriuchi T., Ohmura S.D., Moriuchi-Kawakami T. (2018). Chirality Induction in Bioorganometallic Conjugates. Inorganics.

[58] Byun B.J., Song I.K., Chung Y.J., Ryu K.H., Kang Y.K. (2010). Conformational preferences of X-Pro sequences: Ala-Pro and Aib-Pro motifs. J. Phys. Chem. B.

[59] Martin V., Legrand B., Vezenkov L.L., Berthet M., Subra G., Calmès M., Bantignies J.-L., Martinez J., Amblard M. (2015). Turning Peptide Sequences into Ribbon Foldamers by a Straightforward Multicyclization Reaction. Angew. Chem. Int. Ed..

[60] Metrano A.J., Abascal N.C., Mercado B.Q., Paulson E.K., Hurtley A.E., Miller S.J. (2017). Diversity of Secondary Structure in Catalytic Peptides with β-Turn-Biased Sequences. J. Am. Chem. Soc..

[61] Schrödinger E. (2014). MacroModel.

[62] Mohamadi F., Richards N.G.J., Guida W.C., Liskamp R., Lipton M., Caufield C., Chang G., Hendrickson T., Still W.C. (1990). Macromodel—An integrated software system for modeling organic and bioorganic molecules using molecular mechanics. J. Comput. Chem..

[63] Frisch M.J., Trucks G.W., Schlegel H.B., Scuseria G.E., Robb M.A., Cheeseman J.R., Scalmani G., Barone V., Petersson G.A., Nakatsuji H. (2016). Gaussian 16, Revision, C.01.

[64] Marenich A.V., Cramer C.J., Truhlar D.G. (2009). Universal Solvation Model Based on Solute Electron Density and on a Continuum Model of the Solvent Defined by the Bulk Dielectric Constant and Atomic Surface Tensions. J. Phys. Chem. B.

[65] Koch U., Popelier P.L.A. (1995). Characterization of C-H-O Hydrogen Bonds on the Basis of the Charge Density. J. Phys. Chem..

[66] Popelier P.L.A. (1998). Characterization of a dihydrogen bond on the basis of the electron density. J. Phys. Chem. A.

[67] Caramiello A.M., Bellucci M.C., Cristina G., Castellano C., Meneghetti F., Mori M., Secundo F., Viani F., Sacchetti A., Volonterio A. (2023). Synthesis and Conformational Analysis of Hydantoin-Based Universal Peptidomimetics. J. Org. Chem..

[68] Kong J., Yu S. (2007). Fourier transform infrared spectroscopic analysis of protein secondary structures. Acta Biochim. Biophys. Sin..

[69] Vincenzi M., Mercurio F.A., Leone M. (2021). NMR Spectroscopy in the Conformational Analysis of Peptides: An Overview. Curr. Med. Chem..

[70] Vass E., Hollósi M., Besson F., Buchet R. (2003). Vibrational spectroscopic detection of beta- and gamma-turns in synthetic and natural peptides and proteins. Chem. Rev..

[71] Ramos S., Thielges M.C. (2019). Site-Specific 1D and 2D IR Spectroscopy to Characterize the Conformations and Dynamics of Protein Molecular Recognition. J. Phys. Chem B..

[72] Arunan E., Desiraju G.R., Klein R.A., Sadlej J., Scheiner S., Alkorta I., Clary D.C., Crabtree R.H., Dannenberg J.J., Hobza P. (2011). Defining the hydrogen bond: An account (IUPAC Technical Report). Pure Appl. Chem..

[73] Fornaro T., Burini D., Biczysko M., Barone V. (2015). Hydrogen-bonding effects on infrared spectra from anharmonic computations: Uracil-water complexes and uracil dimers. J. Phys. Chem. A.

[74] Wagner G., Pardi A., Wuethrich K. (1983). Hydrogen bond length and proton NMR chemical shifts in proteins. J. Am. Chem. Soc..

[75] Newberry R.W., Raines R.T. (2019). Secondary Forces in Protein Folding. ACS Chem. Biol..

[76] Llinás M., Klein M.P. (1975). Solution conformation of the ferrichromes. VI. Charge relay at the peptide bond. Proton magnetic resonance study of solvation effects on the amide electron density distribution. J. Am. Chem. Soc..

[77] Stevens E.S., Sugawara N., Bonora G.M., Toniolo C. (1980). Conformational analysis of linear peptides. 3. Temperature dependence of NH chemical shifts in chloroform. J. Am. Chem. Soc..

[78] Kessler H. (1982). Conformation and Biological Activity of cyclic Peptides. Angew. Chem. Int. Ed..

[79] Iqbal M., Balaram P. (1982). Aggregation of apolar peptides in organic solvents. Concentration dependence of 1H-nmr parameters for peptide NH groups in 310 helical decapeptide fragment of suzukacillin. Biopolymers.

[80] Vijayakumar E.K.S., Balaram P. (1983). Stereochemistry of a-aminoisobutyric acid peptides in solution. Helical conformations of protected decapeptides with repeating Aib-L-Ala and Aib-L-Val sequences. Biopolymers.

[81] Andersen N.H., Neidigh J.W., Harris S.M., Lee G.M., Liu Z., Tong H. (1997). Extracting Information from the Temperature Gradients of Polypeptide NH Chemical Shifts. 1. The Importance of Conformational Averaging. J. Am. Chem. Soc..

[82] Baxter N.J., Williamson M.P. (1997). Temperature dependence of 1H chemical shifts in proteins. J. Biomol. NMR.

[83] Cierpicki T., Otlewski J. (2001). Amide proton temperature coefficients as hydrogen bond indicators in proteins. J. Biomol. NMR.

[84] Cierpicki T., Zhukov I., Byrd R.A., Otlewski J. (2002). Hydrogen bonds in human ubiquitin reflected in temperature coefficients of amide protons. J. Magn. Reson..

[85] Pignataro M.F., Herrera M.G., Dodero V.I. (2020). Evaluation of Peptide/Protein Self-Assembly and Aggregation by Spectroscopic Methods. Molecules.

[86] Rogers D.M., Jasim S.B., Dyer N.T., Auvray F., Réfrégiers M., Hirst J.D. (2019). Electronic Circular Dichroism Spectroscopy of Proteins. Chem.

[87] Moriuchi T. (2022). Helical Chirality of Ferrocene Moieties in Cyclic Ferrocene-Peptide Conjugates. Chem. Eur. J..

[88] Barth N.D., Subiros-Funosas R., Mendive-Tapia L., Duffin R., Shields M.A., Cartwright J.A., Henriques S.T., Sot J., Goñi F.M., Lavilla R. (2020). A fluorogenic cyclic peptide for imaging and quantification of drug-induced apoptosis. Nat. Commun..

[89] Li L., Thomas R.M.M., Suzuki H., de Brabander J.K., Wang X., Harran P.G. (2004). A small molecule Smac mimic potentiates TRAIL- and TNFalpha-mediated cell death. Science.

[90] Lyu Z., Yang P., Lei J., Zhao J. (2023). Biological Function of Antimicrobial Peptides on Suppressing Pathogens and Improving Host Immunity. Antibiotics.

[91] Iqbal M., Balaram P. (1981). The 3^10^ helical conformation of the amino terminal decapeptide of suzukacillin. 270 MHz hydrogen-1 NMR evidence for eight intramolecular hydrogen bonds. J. Am. Chem. Soc..

[92] Venkatachalapathi Y.V., Prasad B.V.V., Balaram P. (1982). Conformational analysis of small disulfide loops. Spectroscopic and theoretical studies on a synthetic cyclic tetrapeptide containing cystine. Biochemistry.

[93] Albada B., Metzler-Nolte N. (2017). Highly Potent Antibacterial Organometallic Peptide Conjugates. Acc. Chem. Res..

[94] Soulère L., Bernard J. (2009). Design, solid phase synthesis and evaluation of cationic ferrocenoyl peptide bioconjugates as potential antioxidant enzyme mimics. Bioorganic Med. Chem. Lett..

[95] Xianjiao M., Shengling L., Wenbing M., Jianlong W., Zhiyong H., Duanlin C. (2018). Synthesis and Antioxidant Activities of Ferrocenyl-containing Curcumin Analogues. Lett. Drug Des. Discov..

[96] Kataoka H., Fanali S., Haddad P., Poole C., Riekkola M.-L. (2017). Sample preparation for liquid chromatography. Liquid chromatography: Applications.

[97] Fekete S., Veuthey J.L., Guillarme D. (2012). New trends in reversed-phase liquid chromatographic separations of therapeutic peptides and proteins: Theory and applications. J. Pharm. Biomed. Anal..

[98] Neuhaus C.S., Gabernet G., Steuer C., Hiss J.A., Zenobi R., Schneider G. (2019). Simulated Molecular Evolution for Anticancer Peptide Design. Angew. Chem. Int. Ed..

[99] Shibue M., Mant C.T., Hodges R.S. (2005). Effect of anionic ion-pairing reagent hydrophobicity on selectivity of peptide separations by reversed-phase liquid chromatography. J. Chromatogr. A.

[100] Boyd M.R., Kenneth D.P. (1995). Some practical considerations and applications of the national cancer institute in vitro anticancer drug discovery screen. Drug Dev. Res..

[101] Pascal F., Bedouet L., Baylatry M., Namur J., Laurent A. (2015). Comparative Chemosensitivity of VX2 and HCC Cell Lines to Drugs Used in TACE. Anticancer Res..

[102] Kraatz H.-B., Leek D.M., Houmam A., Enright G.D., Lusztyk J., Wayner D.D.M. (1999). The ferrocene moiety as a structural probe: Redox and structural properties of ferrocenoyl-oligoprolines Fc-Pro*n*-OBzl (*n* = 1–4) and Fc-Pro2-Phe-Obzl. J. Organomet. Chem..

[103] Moriuchi T., Fujiwara T., Hirao T. (2009). β-Turn-structure-assembled palladium complexes by complexation-induced self-organization of ferrocene–dipeptide conjugates. Dalton Trans..

[104] Zong Z., Zhang H., Hao A., Xing P. (2021). The origin of supramolecular chirality in 1-ferrocenyl amino acids. Dalton Trans..

[105] Kovač V., Čakić Semenčić M., Kodrin I., Roca S., Rapić V. (2013). Ferrocene-dipeptide conjugates derived from aminoferrocene and 1-acetyl-1′-aminoferrocene: Synthesis and conformational studies. Tetrahedron.

[106] Toniolo C., Formaggio F., Crisma M., Schoemaker H.E., Kamphuis J. (1994). The *p*-bromobenzamido chromophore as a circular dichroic probe for the assignment of the screw sense of helical peptides. Tetrahedron Asymmetry.

[107] Diemer V., Maury J., Le Bailly B.A.F., Webb S.J., Clayden J. (2017). Dibenzazepinyl ureas as dual NMR and CD probes of helical screw-sense preference in conformationally equilibrating dynamic foldamers. Chem. Commun..

[108] Prasad B.V., Balaram P. (1984). The stereochemistry of peptides containing alpha-aminoisobutyric acid. CRC Crit. Rev. Biochem..

[109] Wüthrich K. (1986). NMR of Proteins and Nucleic Acids.

[110] Gellman S.H., Dado G.P., Liang G.-B., Adams B.R. (1991). Conformation-directing effects of a single intramolecular amide-amide hydrogen bond: Variable-temperature NMR and IR studies on a homologous diamide series. J. Am. Chem. Soc..

[111] Pardi A., Wagner G., Wuthrich K. (1983). Protein conformation and proton nuclear-magnetic-resonance chemical shifts. Eur. J. Biochem..

[112] Omar M.M., Abd El-Halim H.F., Khalil E.A.M. (2017). Synthesis, characterization, and biological and anticancer studies of mixed ligand complexes with Schiff base and 2,2′-bipyridine. Appl. Organomet. Chem..

[113] Steeneck C., Kinzel O., Anderhub S., Hornberger M., Pinto S., Morschhaeuser B., Albers M., Sonnek C., Czekańska M., Hoffmann T. (2021). Discovery and optimization of substituted oxalamides as novel heme-displacing IDO1 inhibitors. Bioorg. Med. Chem. Lett..

[114] Saito F., Schreiner P.R. (2020). Determination of the Absolute Configurations of Chiral Alkanes-An Analysis of the Available Tools. Eur. J. Org. Chem..

[115] Wolf C., Bentleya K.W. (2013). Chirality sensing using stereodynamic probes with distinct electronic circular dichroism output. Chem. Soc. Rev..

